# Programmable RNA *N* ^6^,2´‐O‐Dimethyladenosine Editing

**DOI:** 10.1002/advs.74815

**Published:** 2026-03-13

**Authors:** Yang Li, Xiangmin Tan, Yaran Liu, Yongquan He, Bo Yuan, Ping Wang, Guangzhi Ma, Mengzhe Guo, Jian Zhou, Qiang Sun

**Affiliations:** ^1^ Genetic Diseases Key Laboratory of Sichuan Province Department of Medical Genetics Department of Laboratory Medicine School of Medicine Sichuan Academy of Medical Sciences & Sichuan Provincial People's Hospital University of Electronic Science and Technology of China Chengdu China; ^2^ Center For RNA Medicine and International School of Medicine the Fourth Affiliated Hospital of School of Medicine International Institutes of Medicine Zhejiang University Yiwu China; ^3^ Institute of Medical Artificial Intelligence Binzhou Medical College Yantai China; ^4^ Institute of Artificial Intelligence Beihang University Haidian Beijing China; ^5^ Beijing Advanced Innovation Center for Future Blockchain and Privacy Computing Beihang University Haidian Beijing China; ^6^ Department of Thoracic Surgery and Institute of Thoracic Oncology, West China Hospital Sichuan University Chengdu China; ^7^ School of Pharmacy Xuzhou Medical University Xuzhou China

**Keywords:** CRISPR‐Cas13, *N* ^6^,2´‐O‐dimethyladenosine, RNA editing, RNA modification

## Abstract

*N* ^6^,2’‐O‐dimethyladenosine (m^6^Am) is a prevalent RNA modification located at the first transcribed nucleotide adjacent to the 5′ cap of mRNAs, where it has been implicated in gene regulation. However, the lack of methods for precise, transcript‐specific manipulation of m^6^Am has limited its functional dissection. Here, we develop a programmable RNA‐editing platform, termed Targeted m^6^ Am Methylation (TAmM), that enables site‐specific installation of m^6^Am on selected cellular transcripts. TAmM is engineered by fusing the catalytically inactive RfxCas13d (dCasRx) with the m^6^Am methyltransferase PCIF1, allowing guided deposition of m^6^Am at cap‐proximal nucleotides. Using TAmM, we achieve efficient and specific m^6^Am installation at single‐nucleotide resolution, as validated by LC‐MS/MS. Targeted m^6^Am editing does not alter steady‐state mRNA abundance but modulates protein output in a transcript‐dependent manner. Mechanistically, m^6^Am installation enhances polysome association, indicating a direct role in translational regulation. Functional interrogation demonstrates that cap‐proximal m^6^Am deposition on *CTNNB1* increases *β*‐catenin protein expression, promoting cell proliferation, clonogenicity, and migration in cancer cell models. Importantly, TAmM exhibits high fidelity, with negligible effects on the global m^6^Am landscape, transcriptome, or proteome. Our study establishes TAmM as a precise and versatile platform for programmable m^6^Am manipulation and reveals transcript‐specific roles of m^6^Am in gene regulation.

## 1 Introduction

Dynamic chemical modifications to RNA species play fundamental roles in regulating gene expression [1, 2]. *N* ^6^‐methyladenosine (m^6^A) is the most abundant and prevalent internal mRNA modification in eukaryotic cells [1]. *N* ^6^,2´‐O‐dimethyladenosine (m^6^Am), which has a chemical structure similar to m^6^A, is another abundant mRNA modification that occurs adjacent to the *N* ^7^‐methylguanosine (m^7^G) cap structure of mRNA [3]. In eukaryotic mRNAs, the first transcribed nucleotide next to the m^7^G cap is prone to be 2´‐O‐methylated by cap methyltransferase (MTase) CMTR1 [4]. If this first nucleotide is an adenosine, a portion of these 2´‐O‐methyladenosine (Am) residues can be further methylated at the *N* ^6^ position to yield the m^6^Am modification [4]. Several studies have shown that the fat mass and obesity‐associated protein (FTO), a well‐known m^6^A demethylase, can also catalyze the demethylation of the *N* ^6^‐methyl group of m^6^Am [5, 6]. To date, phosphorylated CTD interacting factor 1 (PCIF1) is the only methyltransferase (MTase) identified to catalyze the m^6^Am modification in eukaryotic mRNAs [7, 8, 9, 10]. Recent studies show that m^6^Am plays critical roles in various physiological and pathological processes [11, 12, 13, 14]. However, current studies of m^6^Am largely rely on global knockdown or overexpression of its modifying enzymes, which inevitably perturbs the global epitranscriptome. Thus, tools that enable the precise editing of m^6^Am in specific mRNAs would pave the way for functionally interrogating the causal relationships between a specific m^6^Am modification and its associated phenotype.

Cas13 is a class 2 type VI CRISPR‐Cas endonuclease that exclusively targets and cleaves single‐stranded RNA [15]. The Cas13 family has been widely utilized to knockdown endogenous mRNAs because of its high efficiency and flexibility in guide RNA (gRNA) design [16, 17, 18, 19, 20]. RfxCas13d (previously named CasRx), an ortholog of CRISPR‐Cas13d with a relatively small molecular size and robust targeting efficiency, has been applied to target and degrade mRNAs in vivo and effectively rescue disease phenotypes in animal models [21, 22, 23]. However, the collateral cleavage activity of the CRISPR‐Cas13 system raises concerns regarding its application for targeting and degrading endogenous RNAs in mammalian cells [24, 25, 26]. Once the Cas13 endonuclease is activated through interaction with the target RNA, which is complementary to the gRNA, it subsequently cleaves and degrades neighboring RNAs in a non‐specific manner [27, 28]. Nonetheless, the catalytically inactive Cas13 (dead Cas13, dCas13), which retains robust RNA‐targeting capability, serves as a programmable platform to target RNA.

Recently, several molecular tools used to edit RNA modifications have been established by conjugating dCas13 proteins with various effector proteins. In particular, fusion of dCas13 with METTL3 successfully installs m^6^A in specific transcripts, further affecting their stability and translation [29, 30, 31]. Conversely, site‐specific demethylation of m^6^A can be accomplished by fusing dCas13 with FTO or AlkB homolog 5 (ALKBH5) [30, 31]. REMOVER is a programmable platform for the targeted demethylation of *N* ^1^‐methyladenosine (m^1^A) modification, using the catalytically inactive CasRx (dCasRx) as the RNA‐targeting module and AlkB homolog 3 (ALKBH3) as the effector protein [32]. Targeted removal of m^1^A in certain transcripts results in their increased stability, indicating a potential role of m^1^A in inducing RNA degradation. Furthermore, the introduction of chemical‐ or light‐inducible methods into these dCas13‐based systems enables conditional editing of diverse RNA modifications [33, 34, 35, 36]. However, approaches that enable precise manipulation of RNA m^6^Am modification remain to be established.

## 2 Methods

### 2.1 Plasmid Constructions

To generate PCIF1‐dCasRx or dCasRx‐PCIF1 constructs, fragments of PCIF1 and dCasRx were cloned with indicated primers using Phusion Hot Start II High Fidelity PCR Mix (Thermo Fisher Scientific). The vector pXR001‐EF1a‐CasRx (Addgene plasmid no. 109049) was also linearized with the indicated primers using Platinum SuperFi II DNA polymerase (Thermo Fisher Scientific). Then the PCR products were fused to the linearized vector through homologous recombination using the ClonExpress Ultra One Step Cloning Kit (Vazyme). For PCIF1 and PCIF1^N553A^ fused with dCasRx constructs, the RNA m^6^Am methyltransferase domain of PCIF1 was cloned from the cDNA of HEK293T cells. The MTase‐impaired PCIF1^N553A^ was generated with the Mut Express II Fast Mutagenesis Kit V2 (Vazyme). Then PCIF1 or PCIF1^N553A^ was fused to the N‐ or C‐terminus of the dCasRx fragment through homologous recombination using the MultiF Seamless Assembly Mix (Abclonal). The CasRx gRNAs were golden‐gate cloned into the pXR003‐CasRx‐gRNA (Addgene plasmid no. 109053) with BbsI‐HF restriction enzyme (NEB) and T4 DNA ligase (NEB), which had constitutive gRNA expression driven by the hU6 promoter. We amplified these plasmids using the DH5α (Tolobio) competent cells. The dCasRx (Addgene plasmid no. 109050) and CasRx gRNA cloning backbone (Addgene plasmid no. 109053) were purchased from Addgene.

### 2.2 gRNA Design and Optimization

Since the 5´ cap and Am together serve as an obligatory substrate for PCIF1, we designed gRNAs positioned within ∼50 nt 3´ to the first adenosine of specified mRNAs. We recommend evaluating 2–3 gRNAs per target mRNA to identify the most effective configuration via m^6^Am‐RIP‐RT‐qPCR. The gRNAs were 30 nt in length throughout the study. All the gRNA spacer sequences were available in Table S1.

### 2.3 Cell Culture

Cells were cultured with Dulbecco's Modified Eagle's Medium (Gibco) supplemented with 10% fetal bovine serum (ExCell). Plasmocin Prophylactic (Invivogen) was added to the culture medium at a concentration of 5 µg/mL to prevent mycoplasma contamination. Cells were cultured at 37°C and 5% CO_2_ in a humidified atmosphere.

### 2.4 Transfection

Cells were cultured and passaged in the 12‐well plates until they reached approximately 80% confluency. Cells were co‐transfected with indicated constructs and corresponding gRNA plasmids at a mass ratio of 1:1 using Lipofectamine 2000 (Thermo Fisher Scientific) according to the manufacturer's instructions. After 48 h of transfection, cells were washed with PBS and followed by subsequent experiments.

### 2.5 m^6^Am‐RIP‐RT‐qPCR

Cells were first transfected with plasmids for 48 h. Next, the total RNA was extracted using TRIzol reagent (Thermo Fisher Scientific). Then 50 µg total RNA was fragmented to 200 nt at 94°C for 4 min using the Magnesium RNA Fragmentation Module (NEB, E6150S). The RNA fragments were purified and concentrated by RNA Clean & Concentrator‐25 spin column (Zymo Research, R1018). 2% of the fragmented RNA was saved as input. Then, the fragmented RNA was incubated with 2 µg m^6^A antibody (SYSY, 202003) and 5 µL RNase Inhibitor (Vazyme) in 1 mL RIP buffer (10 mM Tris‐HCl pH 7.4, 150 mM NaCl, and 0.1% NP‐40) at 4°C for shaking overnight. 20 µL protein A/G beads (MCE, HY‐K0202) were washed twice with RIP buffer, and then incubated in RNA RIP buffer mix and RNase Inhibitor (Vazyme) at 4°C for 4 h. RNA fragments containing m^6^A/m^6^Am on beads were eluted with 200 µL RIP buffer containing 6.7 mM *N* ^6^‐methyladenosine (MCE) and 5 µL RNase Inhibitor (Vazyme), and then purified by RNA Clean & Concentrator‐5 spin column (Zymo Research, R1016), as IP. The m^6^Am level of certain transcripts was subsequently analyzed by RT‐qPCR using specific primers targeting the 5´UTR of target mRNAs.

### 2.6 RNA Isolation and RT‐qPCR

Total RNA was isolated using TRIzol Reagent (Thermo Fisher Scientific), and complementary DNA was generated by HiScript III All‐in‐one RT SuperMix (Vazyme). RT‐qPCR was performed using the ChamQ Universal SYBR qPCR Master Mix (Vazyme) on the CFX96 Real‐Time PCR System (Bio‐Rad). The housekeeping gene *18S* rRNA was used as the internal control. Primers used for RT‐qPCR are listed in Table S2.

### 2.7 Single‐Base Resolution m^6^Am Detection Using Mass Spectrometry

#### 2.7.1 Enrichment of m^6^Am mRNA Fragments

Total RNA was extracted from cells or tissues using TRIzol reagent (Invitrogen) in accordance with the manufacturer's protocol. Approximately 15 µg of total RNA was resuspended in 500 µL of RNase‐free water and subjected to two rounds of poly(A)+ mRNA enrichment using the PolyATtract mRNA Isolation System (Promega, Cat. #Z5310), following the manufacturer's protocol with slight modifications. Each round employed 50 µL of biotinylated oligo(dT) probes. The enriched mRNA was eluted in 100 µL of RNase‐free water and quantified using a NanoDrop One spectrophotometer (Thermo Fisher Scientific). Poly(A)‐enriched mRNA (10 µg) was first treated with 20 U of T4 PNK (NEB, Cat. #M0201S) in T4 ligase buffer (final volume: 20 µL) for 1.5 h at 37°C to phosphorylate 5´‐hydroxyl termini of uncapped RNA fragments. Subsequently, the sample was incubated with 2 U of Terminator 5´‐Phosphate‐Dependent Exonuclease (Lucigen, Cat. #TER51020) at 30°C for 3 h in a 40 µL reaction using 1x Exonuclease Buffer A, thereby removing uncapped and degraded RNA species. To remove the 7‐methylguanosine (m^7^G) cap structure, the sample was incubated with 3 U of mRNA Decapping Enzyme (NEB, Cat. #M0608S) in 1x reaction buffer (final volume: 30 µL) for 2 h at 37°C. The RNA was purified using the RNA Clean & Concentrator‐5 spin column (Zymo Research, R1016) and eluted in 15 µL RNase‐free water and quantified using a NanoDrop One.

#### 2.7.2 Enrichment of Targeted m^6^Am mRNA Fragments by Biotin‐Labeled Probes

Approximately 5 µg of m^6^Am‐enriched mRNA fragments was dissolved in 100 µL of hybridization buffer (6xSSC, Shanghai Sangon Biological Engineering, China). A total of 6 µg of 5´‐biotinylated DNA probes complementary to the m^6^Am‐containing regions were added (5´‐biotin‐GCGGGGCGGACGCGGTCT‐3´), and hybridization was carried out at room temperature for 30 min. DNA–RNA hybrids were precipitated by adding 2.5 volumes of ethanol and 0.1 volumes of 3M sodium acetate (pH 5.2), and the resulting pellet was resuspended in RNase‐free TE buffer (10 mM Tris‐HCl, 0.1 mM EDTA). The purified hybrids were resuspended in 100 µL of digestion buffer (10 mM Tris‐HCl, pH 7.5, 300 mM NaCl, 5 mM EDTA, pH 7.5). To degrade unbound RNA, 0.5 µg of RNase A (Thermo Fisher Scientific, Cat. #EN0531) and 2 U of RNase T1 (Thermo Fisher Scientific, Cat. #EN0541) were added, and the mixture was incubated at 37°C for 15 min. Enzymes were subsequently inactivated by heating the sample at 95°C for 3 min. Next, 50 µL of Streptavidin Magnetic Beads (Thermo Fisher Scientific, Cat. #88816) were added to the mixture and incubated with rotation at room temperature for 15 min to capture biotinylated DNA probes. The hybridized RNA fragments were eluted by incubating the beads with a 50% formamide/RNase‐free water solution at 65°C. This procedure yielded ∼25‐nucleotide RNA fragments harboring m^6^Am modifications, suitable for downstream detection and analysis.

#### 2.7.3 LC‐MS/MS Detection and Analysis

Liquid chromatography‐tandem mass spectrometry (LC–MS/MS) was performed using an EASY‐nLC system (Thermo Fisher Scientific, USA) coupled to an Orbitrap LTQ Lumos mass spectrometer. Separation was achieved using a C18 analytical column (200 mm × 0.75 mm, 3 µm, Welch, China). The mobile phases consisted of 12% acetonitrile with 0.1% formic acid (solvent A) and 98% acetonitrile (solvent B). The gradient elution program was as follows: 0–5 min, 0–70% B; 5–15 min, 70–80% B; 15–20 min, isocratic at 80% B. A 10‐min post‐run equilibration was applied. The flow rate was maintained at 200 nL/min, with an injection volume of 5 µL. The full‐scan resolution was set to 30,000. The ion source temperature was maintained at 250°C, and a spray voltage of 2.5 kV was applied. The instrument operated in positive ion mode. Optimization of ionization and fragmentation conditions was performed using synthetic RNA oligonucleotides of 15, 20, and 25 nucleotides. In‐source dissociation (ISD) energy was optimized to 40 eV, while the collision energy for fragmentation was adjusted to approximately 35 eV to maximize signal intensity for the detection of target RNA fragments. Raw data files generated by Thermo Xcalibur 2.7 were converted to MGF format using MS Converter. Peaks with a signal‐to‐noise ratio (SNR) >10 were selected for compound identification. Sequence alignment and fragment assembly were performed using a previously described software platform (https://Ariadne.riken.jp/html/search.html). Mass tolerance thresholds were set at < 50 ppm for precursor ions and < 400 ppm for fragment ions, following established protocols. All identified sequences were manually validated according to previously reported criteria.

### 2.8 Western Blotting

Cells in 12‐well plates were transfected with indicated plasmids for 48 h. Whole‐cell extracts were generated in RIPA buffer (NCM Biotech) containing Protease Inhibitor Cocktail (NCM Biotech). Lysate was denatured at 100°C for 10 min,10 µL per sample was loaded into a 12‐well SDS‐PAGE 4–12% Bis‐Tris gel (Tsingke Biotech), and gel was wet transferred to a 0.2 µm PVDF membrane (Pall Life Sciences) using a Mini Trans‐Blot Cell Module (Bio‐Rad) for 120 min at 300 mA. Membranes were blocked with 5% skim milk in TBST (TBS + 0.5% Tween‐20) for 1 h at room temperature. Followed by incubation with anti‐GAPDH (Proteintech 60004‐1‐Ig, 1:100 000), anti‐BICD2 (Proteintech 29603‐1‐AP, 1:1000), anti‐*β*‐catenin (Proteintech 51067‐2‐AP, 1:20 000), anti‐*β*‐actin (Proteintech 20536‐1‐AP, 1:10 000), anti‐α‐tubulin (HuaBio HA721913, 1:5000), anti‐*β*‐tubulin (HuaBio ET1602‐4, 1:10 000), anti‐FLAG (Proteintech 66008‐4‐Ig, 1:10 000) in TBST for 1.5 h at room temperature. After washing three times with TBST, membranes were incubated with secondary antibodies, goat anti‐mouse (Proteintech, RGAM001) or goat anti‐rabbit (Proteintech, RGAR001), diluted 1:10 000 in TBST for 1 h at room temperature. The membrane was finally washed three times with TBST, then imaged on the ChemiDoc Touch Imaging System (Bio‐Rad).

### 2.9 Immunofluorescence Staining

Cells were cultured on coverslips in a 12‐well plate for 24 h, and were transfected with indicated plasmids for 24 h. Then cells were fixed with pre‐cooled methanol for 10 min in ‐20°C. The fixed cells were washed with IF buffer (PBS supplemented with 0.1% Triton X‐100) for three times and blocked with 5% BSA in IF buffer for 1 h, and then incubated with mouse anti‐FLAG antibody (Proteintech 66008‐4‐Ig, 1:1000) for 2 h at room temperature. After washing with IF buffer for three times, cells were incubated with anti‐mouse secondary antibody (Thermo Fisher Scientific A‐10680, 1:2000) for 1 h at room temperature and washed with IF buffer for three times. Finally, coverslips were mounted onto slides with antifade reagent containing DAPI (Solarbio) and images were acquired using a fluorescence microscope (Olympus IX83‐FV3000‐OSR).

### 2.10 RNA‐seq and Data Analysis

RNA‐seq libraries were constructed with the NEBNext Ultra RNA Library Prep Kit for Illumina (NEB) according to the manufacturer's guidelines. The quality of the libraries, including size distribution and concentration, was evaluated using the Agilent 4200 Bioanalyzer. Paired‐end 150‐bp sequencing (PE150) was conducted on the Illumina NovaSeq X Plus platform. Raw reads were filtered for adapter removal and low‐quality sequences using fastp (v0.23.4). The filtered reads were aligned to the hg38 reference genome using HISAT2 (v2.2.1), and uniquely mapped reads were processed via featureCounts (v2.0.6) to produce a read count matrix. Differential gene expression analysis was performed with DESeq2 (v1.44.0), and genes with |log_2_(fold change)| >1 and *p* < 0.01 were identified as differentially expressed. Transcripts Per Kilobase Million (TPM) were derived from the read count matrix using a custom Python script for visualization purposes.

### 2.11 m^6^Am‐Exo‐Seq and Data Analysis

#### 2.11.1 mRNA Purification

200 µg of total RNA was subjected to two rounds of Poly(A) mRNA purification using the magnetic mRNA isolation kit from (NEB). 100 µL of oligo d(T) magnetic beads were used per purification. mRNA was eluted in a final volume of 50 µL, in elution buffer.

#### 2.11.2 Cap‐m^6^A RNA Immunoprecipitation

11 µg mRNA was incubated for 5 min at 94°C to generate ∼150 nt‐long fragments using RNA fragmentation module (NEB, E6150S). The stop solution buffer was added to stop the reaction, and RNA fragments were purified with the RNA Clean and Concentrator‐5 (Zymo Research) and eluted in 10 µL of elution buffer. Uncapped and fragmented transcripts were phosphorylated by treating the mRNA with 20U of T4 PNK (NEB) in T4 ligase buffer in a final volume of 20 µL for 1.5 h at 37°C. Then, 2U of Terminator 5´‐Phosphate‐Dependent Exonuclease (Lucigen) was added and volume increased to 40 µL with 1 × Exonuclease buffer A. Samples were treated for 3 h at 30°C to remove phosphorylated (uncapped and sheared) transcripts. The RNA enriched for 5´‐capped transcripts was purified with the RNA Clean and Concentrator‐5 kit (Zymo Research, R1016) and eluted in 15 µL. 3 U of mRNA Decapping Enzyme (NEB, Cat. #M0608S) were added and incubated for 2 h at 37°C to remove the m^7^G cap. RNA was again purified with the RNA Clean and Concentrator‐5 kit (Zymo Research, R1016), eluted in 11 µL and quantified in a NanoDrop One. At this point, approximately 1 µg of uncapped 5´ RNA fragments were obtained. 1 µL RNA fragments (10%) were used as “input”. The rest was diluted to a final volume of 250 µL with reaction buffer (10 mM Tris pH 7.4, 150 mM NaCl, 0.1% Igepal). 100 µL of Protein A/G Dynabeads (Invitrogen) per sample were washed twice with 250 µL of reaction buffer and resuspended in 250 µL of reaction buffer with 2.5 µg of anti‐m^6^A antibody (SYSY, 202003). Beads and antibody were incubated in rotation for 1 h at 4°C and then washed twice with 250 µL of ice‐cold reaction buffer. The beads/antibody slurry was added to the uncapped RNA in a final volume of 500 µL of reaction buffer and incubated in rotation for 2 h at 4°C. Beads were washed twice with 200 µL of reaction buffer at room temperature, twice with 200 µL of low salt buffer (10 mM Tris pH 7.4, 50 mM NaCl, 0.1% Igepal) at room temperature and twice with 200 µL of high salt buffer (10 mM Tris pH 7.4, 500 mM NaCl, 0.1% Igepal). Immunoprecipitated 5´ RNA fragments containing m^6^A on the beads were eluted with 200 µL RIP buffer (10 mM Tris‐HCl pH 7.4, 150 mM NaCl, and 0.1% NP‐40) containing 6.7 mM *N* ^6^‐methyladenosine (MCE) and 5 µL RNase inhibitor (Vazyme) for 15 min at room temperature, and then purified by RNA Clean & Concentrator kit‐5 (Zymo Research, R1016), as “IP”.

#### 2.11.3 Sequencing

Illumina sequencing libraries were prepared using the NEBNext Ultra II Directional RNA Library Prep Kit for Illumina (NEB, Cat. #E7760) according to the manufacturer's instructions. Libraries were sequenced on a NextSeq 500 platform (Illumina) using a single‐end read configuration, generating a minimum of 15 million reads per sample.

#### 2.11.4 Data Analysis

Sequenced reads were initially processed to eliminate adapter sequences and low‐quality bases using fastp (v0.23.4). The resulting high‐quality reads were aligned to human ribosomal RNA reference sequences with bowtie2 (v2.5.4) to filter out rRNA‐mapped reads. The remaining reads were subsequently aligned to the human genome (hg38) using HISAT2 (v2.2.1) with the parameters –no‐unal –rna‐strandness R. Alignment files in SAM format were sorted and converted into BAM format using samtools (v1.21). To facilitate visualization, normalized bigWig tracks were created from BAM files using bamCoverage from the deepTools suite (v3.5.4) with the options: ‐bs 20, ‐normalizeUsing BPM, and ‐skipNAs. Biological replicates were merged to produce average signal tracks using bigwigCompare from the deepTools suite. Both metagene plots and heatmaps were generated based on these bigWig files, using a combination of deepTools suite and custom scripts. A SAF file was generated using in‐house script, which defined the transcription start site (TSS) regions‐from TSS to +300 base pairs‐for all coding transcripts based on annotations from the refTSS database (http://reftss.clst.riken.jp/). Using this SAF file, reads overlapping TSS regions were quantified with featureCounts (v2.0.6). The resulting count matrix was analyzed in R using the edgeR package (v3.40.0), where counts per million (CPM) were calculated. Normalization factors were computed, and biological dispersion was estimated. To identify m^6^Am‐enriched transcripts, replicates were normalized against corresponding input samples, and transcripts with FDR < 0.01 were classified as significantly enriched.

### 2.12 DIA Proteomics and Data Analysis

#### 2.12.1 Protein Extraction and Tryptic Digestion

Cells were lysed in RIPA lysis buffer supplemented with protease and phosphatase inhibitors for 30 min, then sonicated using a grinding instrument at 4°C. The lysates were centrifuged at 13,800 × g for 15 min to remove debris, and protein in the supernatant was quantified using a BCA protein assay. To the protein solution, 20 mM Tris (2‐chloroethyl) phosphate (TCEP) was added and incubated at 60°C for 1 h, followed by the addition of 200 mM chloroacetamide (CAA) for 30 min at room temperature in darkness. Proteins were precipitated with ice‐cold acetone at ‐20°C for 4 h, then the pellet was air‐dried and resuspended in 150 µL of 0.1 M ammonium bicarbonate (ABB). Proteins were digested with trypsin (enzyme‐to‐substrate ratio of 1:25) at 37°C for 16 h, desalted using SOLA HRP cartridges, and vacuum‐dried with a SpeedVac.

#### 2.12.2 Nano‐LC‐MS/MS Analysis

Peptide samples were analyzed using an EASY‐nLC 1200 system coupled with Q Exactive HF Orbitrap mass spectrometry (Thermo Fisher). The analytical column was a C18 nano‐capillary (25 cm × 75 mm, 1.9 µm). Peptides were re‐dissolved in mobile phase A (Water/ACN/formic acid, 98/2/0.1, *v/v*). The gradient was as follows: 0–5 min, 5–10% B; 5–55 min, 10–28% B; 55–58 min, 28–40% B; 58–63 min, 40–95% B; and 63–75 min, 95% B, with a flow rate of 350 nL/min. Mass spectrometry operated in data‐independent acquisition (DIA) mode, with an *m/z* range of 350–1,500 Da at a resolution of 60,000 for MS1. The automatic gain control (AGC) was set to 3 ×1 0^6^, with a maximum ion injection time of 50 ms. MS2 acquisition used higher‐energy collision dissociation (HCD) at 30 eV, with a resolution of 30,000, a loop count of 25, an MSX count of 1, an isolation window of 8.3 *m/z*, and a fixed maximum mass of 200 *m/z*.

#### 2.12.3 MS Database Search and Analysis

The MS database search was conducted using DIA‐NN (v1.9) against UniProt human protein database (release‐2024_03). The parameters were set as follows: the number of missed cleavages was 3, the peptide length range was 5–100, the precursor charge range was 1–7, the precursor *m/z* range was 250–2,000, and the fragment ion m/z range was 300–2,000. Quantification of identified peptides was determined by averaging the chromatographic fragment ion peak areas across all reference spectral libraries. The analysis in this study focused on proteins identified in more than 70% of the samples. Missing values were treated as NaN for downstream analysis. *p* value between two groups was derived by two‐sided paired *t*‐test, proteins with |log_2_(fold change)| >1 and *p* < 0.01 were identified as differentially expressed.

### 2.13 Generation of Stable Cell Line

Lentiviral particles were produced in HEK293T cells by transient transfection. HEK293T cells were cultured in 10‐cm dishes and transfected with the indicated plasmids using polyethyleneimine (Yeasen Biotechnology, 40816ES02) at 70–80% confluence. The lentiviral transfer plasmid, psPAX2, and pMD2.G were co‐transfected at a ratio of 4:3:2. Culture medium was changed after 12 h post‐transfection. Viral supernatants were harvested at 48 h, clarified by centrifugation and filtration through a 0.45‐µm filter, and subsequently concentrated by lentivirus concentration solution (Yeasen Biotechnology, 41101ES50). After incubation for 12 h, viral particles were centrifuged at 4°C and 4,000 × g for 1 h, then the pellets were resuspended in PBS, aliquoted, and stored at ‐80°C. SW480 and HeLa cells were transduced with the indicated lentiviruses, and 48 h later, cells were selected with 2 µg/mL puromycin (Yeasen Biotechnology, 727136ES03) to enrich for successfully transduced populations.

### 2.14 RNA Stability Assay

For RNA stability assay, SW480 and HeLa stable cells expressing the editor and corresponding gRNA were seeded into 6‐well plates, and 12 h later, treated with 15 µg/mL actinomycin D (MedChemExpress, HY‐17559) to inhibit transcription. Cells were harvested at 0, 4, 8, 12, and 24 h after treatment. Total RNA was extracted and reverse transcribed into cDNA, the expression levels of indicated genes were quantified by RT–qPCR.

### 2.15 Cell Proliferation Assay

Cell viability was measured using the CCK‐8 assay (Yeasen Biotechnology, 40203ES80). SW480 and HeLa stable cells expressing the editor and corresponding gRNA were seeded into 96‐well plates at 1,000 cells per well. After the indicated culture periods, 10 µL of CCK‐8 reagent was added to each well and incubated for 2 h at 37°C. Absorbance at 450 nm was recorded using a microplate reader.

### 2.16 Colony Formation Assay

Growing cells were seeded into a 6‐well plate (1,000 cells/well), incubated and observed daily. When an adequate clone appeared, cells were fixed with 4% paraformaldehyde (Biosharp, BL539A), then treated for 30 min with 0.1% crystal violet (Sangon Biotech, A600331). A digital camera was used to photograph and count cell clones.

### 2.17 Cell Migration Assay

The transwell migration assay was conducted using 24‐well transwell chambers (Corning, 3422). SW480 and HeLa stable cells expressing the editor and corresponding gRNA were resuspended at 2 × 10^5^ cells in 200 µL of serum‐free media and seeded into the upper chamber, 700 µL of DMEM with 20% FBS was added to the lower chamber. After indicated incubation, cells that had migrated to the lower surface of the filter were carefully wiped off and fixed with 4% paraformaldehyde (Biosharp, BL539A) for 30 min, followed by staining with 0.1% crystal violet (Sangon Biotech, A600331) for 30 min. Subsequently, the migrated cells were visualized under a fluorescence microscope.

### 2.18 Polysome Profiling

SW480 stable cells expressing editor and corresponding gRNA were harvested at 80% confluency, washed twice with ice‐cold PBS and lysed in polysome profiling lysis buffer (10 mM Tris HCl pH 7.5, 5 mM MgCl2, 100 mM KCl, 100 µg/ml cycloheximide, 2 mM dithiothreitol, 1% Triton X‐100). Optical density values at 260 nm (OD260) were measured for each lysate, and equivalent OD260 units were loaded in 15–60% sucrose gradients prepared using Gradient Master (Biocomp). Sucrose gradients were ultracentrifuged (SW55 Ti rotor; Beckman, 40,000 rpm, 2.5 h, 4°C) and fractionated by displacement with 70% sucrose/0.01% bromophenol blue, using BR‐188 Density Gradient Fractionation System (∼375 µl per fraction, totally 12 fractions) equipped with a UV lamp for continuous 254 nm absorbance monitoring. After fractionation, Trizol (Invitrogen, 15596018CN) was immediately added to all fractions. Each fraction was spiked with RLuc RNA to normalize for recovery of RNA from each fraction. RNA was extracted by the Direct‐zol RNA Miniprep Plus Kit (Zymo Research, R2072). cDNA was synthesized by HiScript III All‐in‐one RT SuperMix (Vazyme, R433‐01). RNA levels for each fraction were quantified by RT‐qPCR to map the distribution.

### 2.19 Statistical Analysis

 *p* values were calculated using unpaired two‐sided Student's *t*‐tests on GraphPad Prism 9. *p < 0.05, **p < 0.01, ***p < 0.001. n.s. stands for not significant. The values represent technical replicates, and all the experiments are replicated at least three independent times. Error bars represent mean±s.d.

## 3 Results

### 3.1 Design and Screening of the Programmable m^6^Am Editor

**FIGURE 1:**
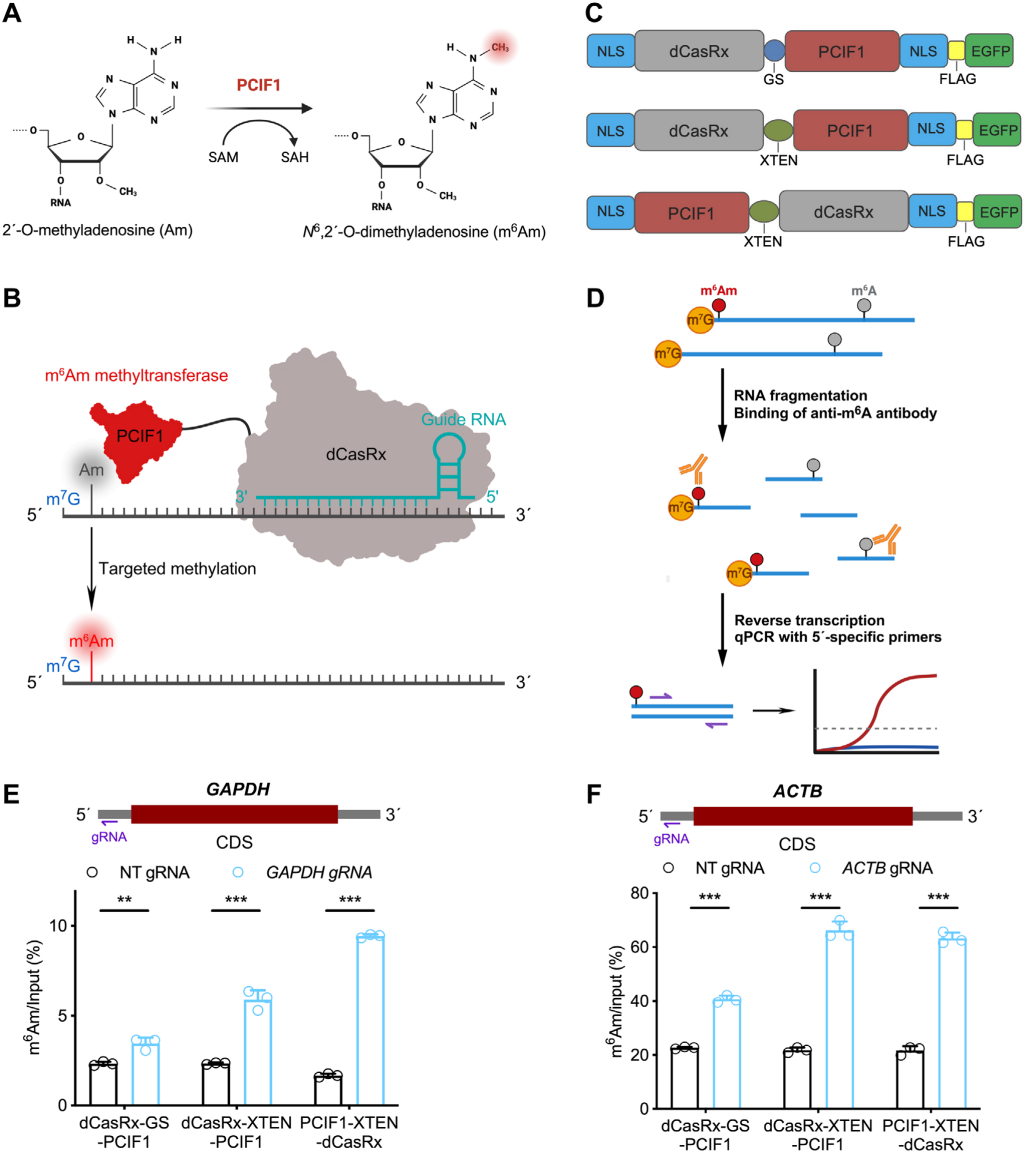
Design and verification of the TAmM editor. (A) PCIF1 catalyzes the final step of m^6^Am methylation by transferring a methyl group from S‐adenosylmethionine (SAM) to the *N* ^6^ position of 2´‐O‐methyladenosine (Am). (B) Fusion of dCasRx and PCIF1 can mediate the gRNA‐dependent installation of m^6^Am into a target mRNA. (C) Construction of three dCasRx and PCIF1 fusions. (D) Schematic illustration of the m^6^A RNA immunoprecipitation followed by RT‐qPCR with specific primers targeting the 5´UTR (m^6^Am‐RIP‐RT‐qPCR). (E, F) Comparison of the m^6^Am abundance in *GAPDH* and *ACTB* mRNAs after co‐transfection of dCasRx‐GS‐PCIF1, dCasRx‐XTEN‐PCIF1, or PCIF1‐XTEN‐dCasRx constructs and NT‐ or *GAPDH*‐targeting gRNA by m^6^Am‐RIP‐RT‐qPCR. NT, non‐targeting control. Error bars represent the mean ± s.d. (**p < 0.01, ***p < 0.001 by two‐tailed Student's *t*‐test).

We sought to develop a programmable molecular tool that allows for precise m^6^Am editing in specific mRNAs. Thus, we speculated that combining the RNA‐targeting capability of dCas13 with an m^6^Am MTase would enable the programmable deposition of m^6^Am within mRNAs. Since PCIF1 has been identified as the enzyme that catalyzes m^6^A methylation of Am at the 5´‐end of mRNAs (Figure 1A) [7, 8, 9, 10]. Furthermore, PCIF1 predominantly co‐localizes with RNA polymerase II (RNAP II) in the cell nucleus [37], and the PCIF1‐mediated m^6^Am methylation is a cap‐specific process that occurs co‐transcriptionally [7, 8, 9, 10]; we therefore reasoned that PCIF1 would be suitable as the effector protein within the m^6^Am editing tool. Among all the Cas13 members, CasRx has been shown to bind and degrade target RNAs with high efficiency and specificity in the cell nucleus [17, 35, 38]. Therefore, we chose dCasRx as the RNA‐targeting component of the m^6^Am editing tool to ensure its RNA‐targeting efficiency in the nucleus. Together, we propose that fusion of PCIF1 and dCasRx could enable the programmable installation of m^6^Am into a defined mRNA in a gRNA‐dependent manner (Figure 1B). To obtain the most efficient m^6^Am editing tool, we engineered three constructs with a nuclear localization signal (NLS) flanking both termini and a FLAG‐EGFP tag located at the carboxy terminus: 1) PCIF1 was fused to the carboxy terminus of dCasRx linked by a GS linker; 2) PCIF1 was fused to the carboxy terminus of dCasRx linked by a flexible 16‐residue XTEN linker [39]; and 3) PCIF1 was fused to the amino terminus of dCasRx linked by an XTEN linker (Figure 1C). Western blotting results verified that all three FLAG‐tagged dCasRx‐PCIF1 fusions were successfully expressed in HEK293T cells (Figure S1A). Fluorescence imaging of cells transfected with all three fusions harboring C‐terminal EGFP confirmed that NLS‐tagged fusions localized in the nucleus (Figure S1B).

Next, we attempted to evaluate the editing efficiency of the three constructs by targeting the endogenous *GAPDH* and *ACTB* mRNAs in HEK293T cells, which are partially methylated at the first adenosine (Figure S2A,B) [40]. To determine the m^6^Am level, we first performed RNA fragmentation and RNA immunoprecipitation (RIP) with an m^6^A antibody that indiscriminately binds to both m^6^A and m^6^Am modifications [41, 42], then the m^6^Am level of the targeted mRNA was detected by quantitative PCR with reverse transcription (RT‐qPCR) using specific primers targeting the 5´ untranslated region (5´UTR) (Figure 1D). As a result, dCasRx‐XTEN‐PCIF1 demonstrated significantly higher editing efficiency than dCasRx‐GS‐PCIF1 for both transcripts, indicating that the XTEN linker outperforms the short GS linker (Figure 1E,F). Notably, while dCasRx‐XTEN‐PCIF1 and PCIF1‐XTEN‐dCasRx showed comparable editing efficiency for *ACTB* mRNA, the latter construct exhibited superior performance for *GAPDH* mRNA (Figure 1E,F). Overall, these results established PCIF1‐XTEN‐dCasRx, referred to as Targeted m^6^Am Methylation (TAmM) hereafter, as the optimal construct for m^6^Am installation in human cells.

### 3.2 Targeted m^6^Am Editing With the TAmM System

**FIGURE 2:**
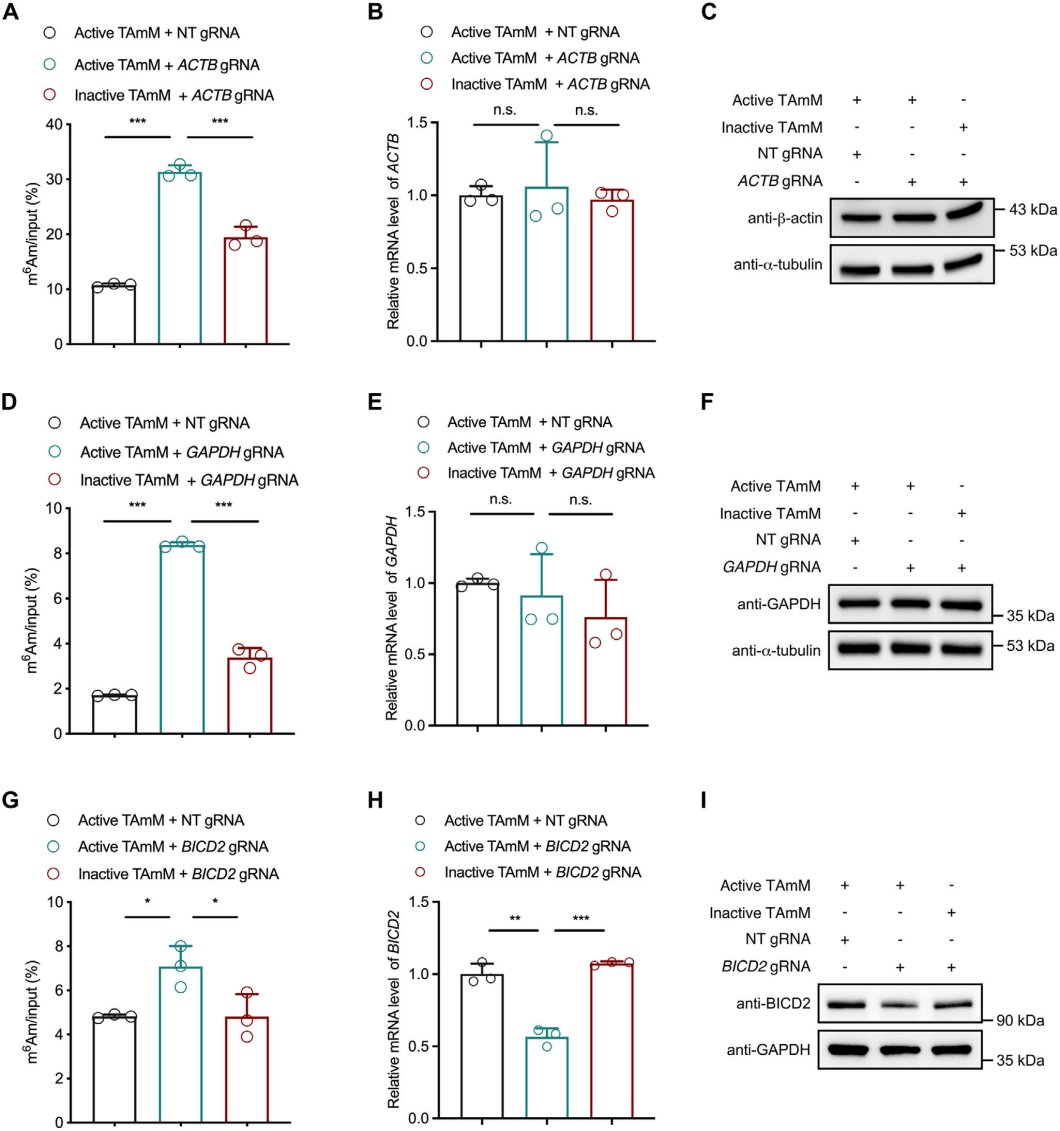
TAmM‐mediated m^6^Am installation of cellular mRNAs and its functional consequences. (A) m^6^Am levels in *ACTB* mRNA quantified by m^6^Am‐RIP‐RT‐qPCR. (B, C) mRNA and protein levels of ACTB measured by RT‐qPCR and western blot, respectively. (D) m^6^Am levels in *GAPDH* mRNA quantified by m^6^Am‐RIP‐RT‐qPCR. (E, F) mRNA and protein levels of GAPDH measured by RT‐qPCR and western blot, respectively. (G) m^6^Am levels in *BICD2* mRNA quantified by m^6^Am‐RIP‐RT‐qPCR. (H, I) mRNA and protein levels of BICD2 measured by RT‐qPCR and western blot, respectively. NT, non‐targeting control. Error bars represent mean ± s.d. (n.s., not significant, *p < 0.05, **p < 0.01, ***p < 0.001 by two‐tailed Student's *t*‐test).

To further evaluate the general applicability of the TAmM editor for targeted m^6^Am installation on endogenous mRNAs, we analyzed the m^6^Am‐seq of HEK293T cells from a previous report [40]. *GAPDH*, *ACTB*, and *BICD2* mRNAs were selected as additional candidates because these transcripts started with adenosine and possessed a modest m^6^Am modification ratio, making them appropriate targets for m^6^Am installation (Figure S2A‐C). Furthermore, to investigate whether TAmM‐mediated m^6^Am installation depends on both the specific gRNA and the MTase activity, we compared the m^6^Am levels among three combinations of non‐targeting/targeting gRNAs and active/inactive TAmM editors using the previously described m^6^Am‐RIP‐RT‐qPCR method. Compared to the non‐targeting gRNA, co‐transfection of the *ACTB*‐targeting gRNA and active TAmM editor resulted in a significant increase in the m^6^Am ratio in *ACTB* mRNA (Figure 2A). Since Asn553 is a conserved residue in the m^6^Am MTase domain of PCIF1 and has been proven to be critical for m^6^Am formation in cells, thus PCIF1^N553A^‐XTEN‐dCasRx serves as an inactive control, referred to as inactive TAmM hereafter [8]. We confirmed that the inactive TAmM editor was successfully expressed in HEK293T cells by western blotting (Figure S3A). Fluorescence imaging further confirmed the nuclear localization of active and inactive TAmM editors (Figure S3B). Notably, co‐expression of the *ACTB*‐targeting gRNA with the inactive TAmM editor, which consists of dCasRx and the catalytic mutant PCIF1^N553A^, did not result in a noticeable elevation of m^6^Am modification in *ACTB* mRNA, demonstrating that MTase activity is necessary for m^6^Am installation (Figure 2A).

Several studies have shown that m^6^Am modification regulates the stability and translation of certain mRNAs [5, 8, 9, 10]. Using the established TAmM editor, we can precisely install m^6^Am into a defined mRNA and subsequently investigate its biological function. Notably, targeted installation of m^6^Am into *ACTB* mRNA did not significantly affect its mRNA expression (Figure 2B). As expected, the protein levels of ACTB were unaffected before or after m^6^Am installation (Figure 2C). Similarly, targeted installation of m^6^Am into the *GAPDH* mRNA showed no significant impact on its mRNA and protein levels (Figure 2D‐F). We reasoned that mRNAs of the two housekeeping genes are decorated with various RNA modifications including m^6^A, m^5^C, and m^1^A [43, 44, 45], thus sole perturbation of the m^6^Am level would not significantly affect their mRNA stability and translation.

We next evaluated the influence of TAmM‐mediated m^6^Am editing on the non‐housekeeping gene *BICD2*. Results showed that the active TAmM editor successfully deposited m^6^Am in *BICD2* mRNA when co‐transfected with the corresponding gRNA, but not with the inactive TAmM editor (Figure 2G). TAmM‐mediated m^6^Am installation caused a 44% decrease in *BICD2* mRNA levels, while the inactive TAmM control remained unaffected (Figure 2H). Moreover, BICD2 protein levels were also downregulated following m^6^Am installation (Figure 2I). These findings are in accordance with a previous study showing that the stability and translation efficiency of *BICD2* mRNA are negatively regulated by PCIF1 [14]. Collectively, these findings suggest that the TAmM editor can install m^6^Am modification in a programmable manner. Importantly, targeted m^6^Am installations have distinct effects on different mRNAs, establishing TAmM as a useful tool to precisely interrogate the causal relationship between a specific m^6^Am modification and its phenotypic outcome.

### 3.3 Detection of Targeted m^6^Am Editing at Single‐nucleotide Resolution With LS‐MS/MS

**FIGURE 3:**
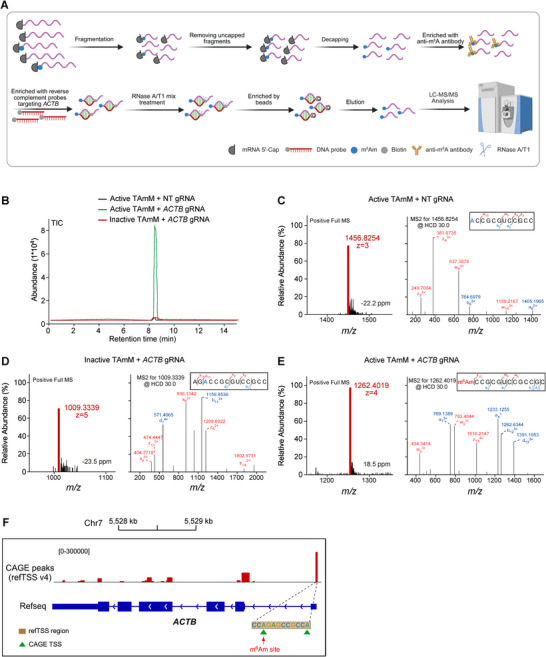
Mass spectrometry confirms site‐specific installation of m^6^Am at the *ACTB* TSS by TAmM. (A) Schematic overview of the RNA enrichment and LC–MS/MS workflow used to detect cap‐proximal m^6^Am at the *ACTB* transcript, including RNA fragmentation, cap selection, antibody‐based enrichment, RNase digestion, and mass spectrometric analysis. (B) Total ion chromatograms (TICs) of RNA fragments from HEK293T cells expressing active TAmM with NT gRNA (black), active TAmM with *ACTB* gRNA (green), and inactive TAmM with *ACTB* gRNA (red). (C–E) The primary, secondary mass spectrum, and sequence analysis based on MS of the targeted fragment in each group. (F) Genomic mapping of the identified m^6^Am site to the annotated *ACTB* transcription start site (TSS), overlapping with CAGE‐seq peaks, confirming precise on‐targeted editing. NT, non‐targeting control.

To biochemically validate the installation of m^6^Am at the intended site, we performed liquid chromatography‐tandem mass spectrometry (LC‐MS/MS) analysis of RNA fragments derived from cells expressing either active or inactive TAmM in combination with targeting or non‐targeting gRNAs, using our previously established enrichment and detection workflow (Figure 3A) [46]. Total ion chromatogram (TIC) analysis revealed a distinct peak at approximately 8.5 min, corresponding to RNA fragments containing the m^6^Am‐modified sequence, which was detected exclusively in the active TAmM + *ACTB* gRNA group (Figure 3B). This peak was absent in both the active TAmM + NT gRNA control and the inactive TAmM + *ACTB* gRNA condition, indicating that m^6^Am installation requires both guide RNA targeting and methyltransferase activity.

Full MS and MS/MS spectra analysis further confirmed the identity and modification status of the detected RNA fragments. In the active TAmM + NT gRNA group and inactive TAmM + *ACTB* gRNA group, the dominant precursor ions (m/z 1456.8254 and 1009.3339, respectively) did not display diagnostic fragmentation patterns indicative of m^6^Am (Figure 3C,D). In contrast, RNA fragments from the active TAmM + *ACTB* gRNA group displayed a prominent precursor ion at m/z 1262.4019 (Figure 3E), and subsequent MS^2^ analysis revealed diagnostic fragment ions consistent with the presence of m^6^Am at the expected cap‐adjacent nucleotide. Notably, a characteristic mass shift corresponding to m^6^Am was observed in the w_10_ ion, and fragment ion mapping unambiguously localized the modification to the target adenosine within the *ACTB* transcript. Importantly, genomic mapping showed that the installed m^6^Am precisely coincides with the annotated transcription start site (TSS) of *ACTB* and overlaps with CAGE‐seq peaks, confirming single‐nucleotide, on‐target editing by TAmM (Figure 3F). Together, these LC‐MS/MS data provide direct biochemical evidence that TAmM installs m^6^Am at the intended site in a gRNA‐dependent and methyltransferase activity dependent manner, addressing a critical need for definitive validation of m^6^Am editing beyond antibody‐based enrichment assays such as m^6^Am‐RIP–qRT‐PCR.

### 3.4 Off‐target Effects of the TAmM System

**FIGURE 4:**
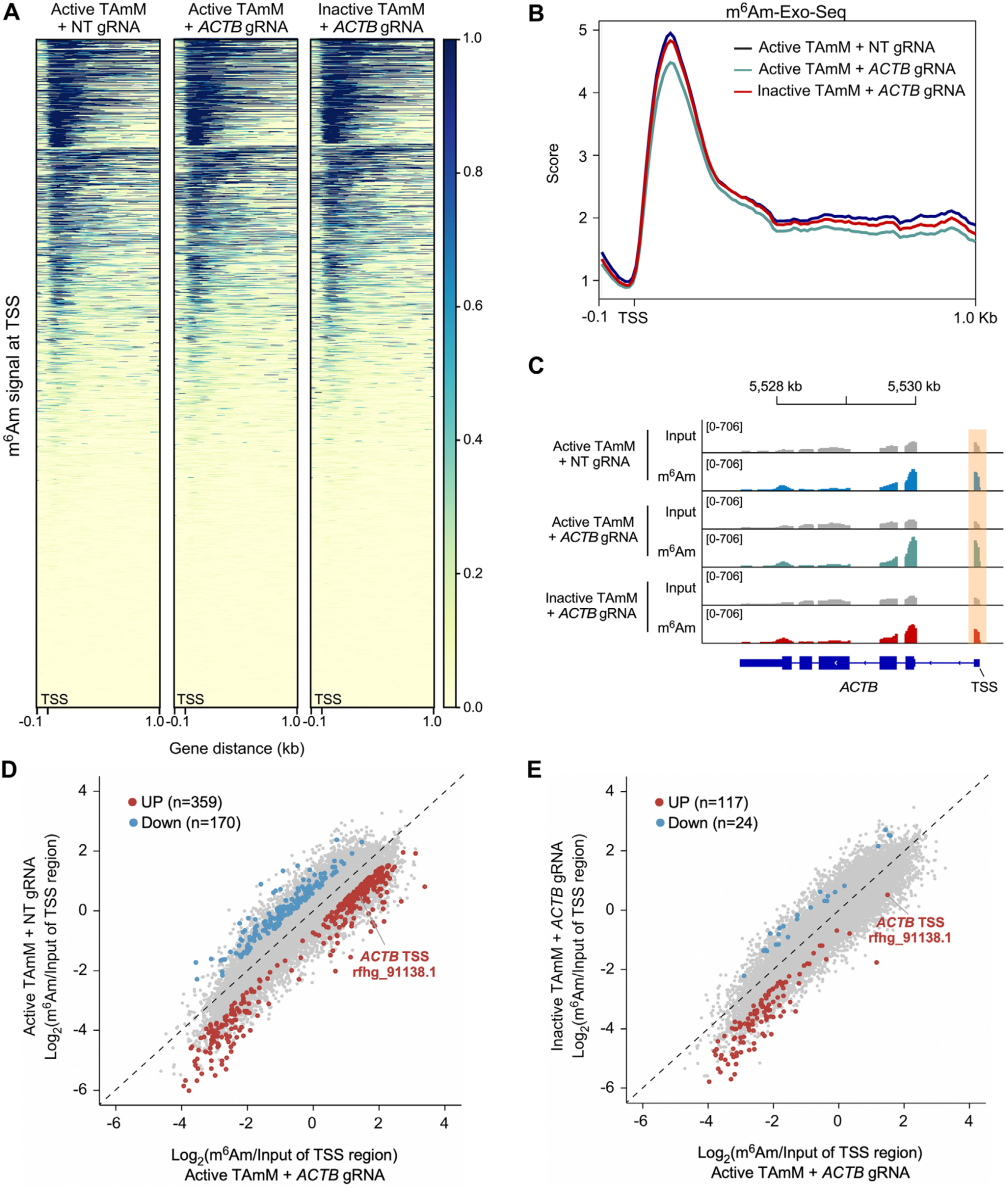
TAmM installs m^6^Am specifically at targeted loci without altering global m^6^Am distribution. (A) Heatmaps showing m^6^Am signal intensities aligned at TSS across the genome in cells treated with active TAmM + NT gRNA, active TAmM + *ACTB* gRNA, and inactive TAmM + *ACTB* gRNA. (B) Aggregated metagene profiles of m^6^Am signal intensities within −100 bp to 1 kb of the TSS across all expressed transcripts. (C) Genome browser tracks showing m^6^Am‐Exo‐Seq signals at the *ACTB* locus. (D) Scatter plot comparing m^6^Am enrichment [log_2_(m^6^Am/Input)] at TSS regions between active TAmM + *ACTB* gRNA and active TAmM + NT gRNA group. (E) Scatter plot comparing m^6^Am enrichment of active TAmM + *ACTB* gRNA with that of inactive TAmM + *ACTB* gRNA. NT, non‐targeting control.

To rigorously evaluate the specificity of our TAmM system, we performed transcriptome‐wide mapping of m^6^Am using m^6^Am‐Exo‐Seq. Heatmap and metagene analyses revealed that the global distribution of m^6^Am signals around the TSS remains largely unchanged across all conditions, including active TAmM with either NT gRNA or *ACTB*‐targeting gRNA, as well as the inactive TAmM control, indicating that TAmM does not disrupt endogenous m^6^Am patterns genome‐wide (Figure 4A,B). Importantly, the m^6^Am enrichment fold at the *ACTB* TSS was significantly higher in the active TAmM + *ACTB* gRNA condition than in the NT gRNA control or the inactive editor condition, confirming that the methylation was both gRNA‐directed and enzymatically dependent (Figure 4C). Scatter plot comparisons between experimental groups further demonstrate that m^6^Am increases were limited to a small number of transcripts, with *ACTB* showing prominent signal (Figure 4D,E). Notably, the inactive TAmM + *ACTB* gRNA group did not induce significant m^6^Am enrichment, reinforcing the high specificity of the active system. Furthermore, we performed m^6^Am‐RIP‐RT‐qPCR to examine the m^6^Am level of several potential off‐target mRNAs (*HMGA2*, *TERF1*, *HIF1A*, *SON*, *CCN2*, and *CCND1*), all of which contain adenosine as their first nucleotide (Figure S4) [40]. After co‐transfection of the *ACTB*‐targeting gRNA and TAmM editor, we observed robust m^6^Am installation into *ACTB* mRNA while the m^6^Am level of most of the potential off‐target mRNAs remained unaffected (Figure S5). However, we did observe some differences between the inactive and active TAmM editors, suggesting a potential off‐target effect dependent on MTase activity (Figure S5). Together, these results provide compelling evidence that TAmM is a highly selective RNA editing platform capable of installing m^6^Am at predetermined sites without inducing widespread off‐target methylation. This specificity supports its potential utility in epitranscriptomic research and therapeutic applications.

**FIGURE 5:**
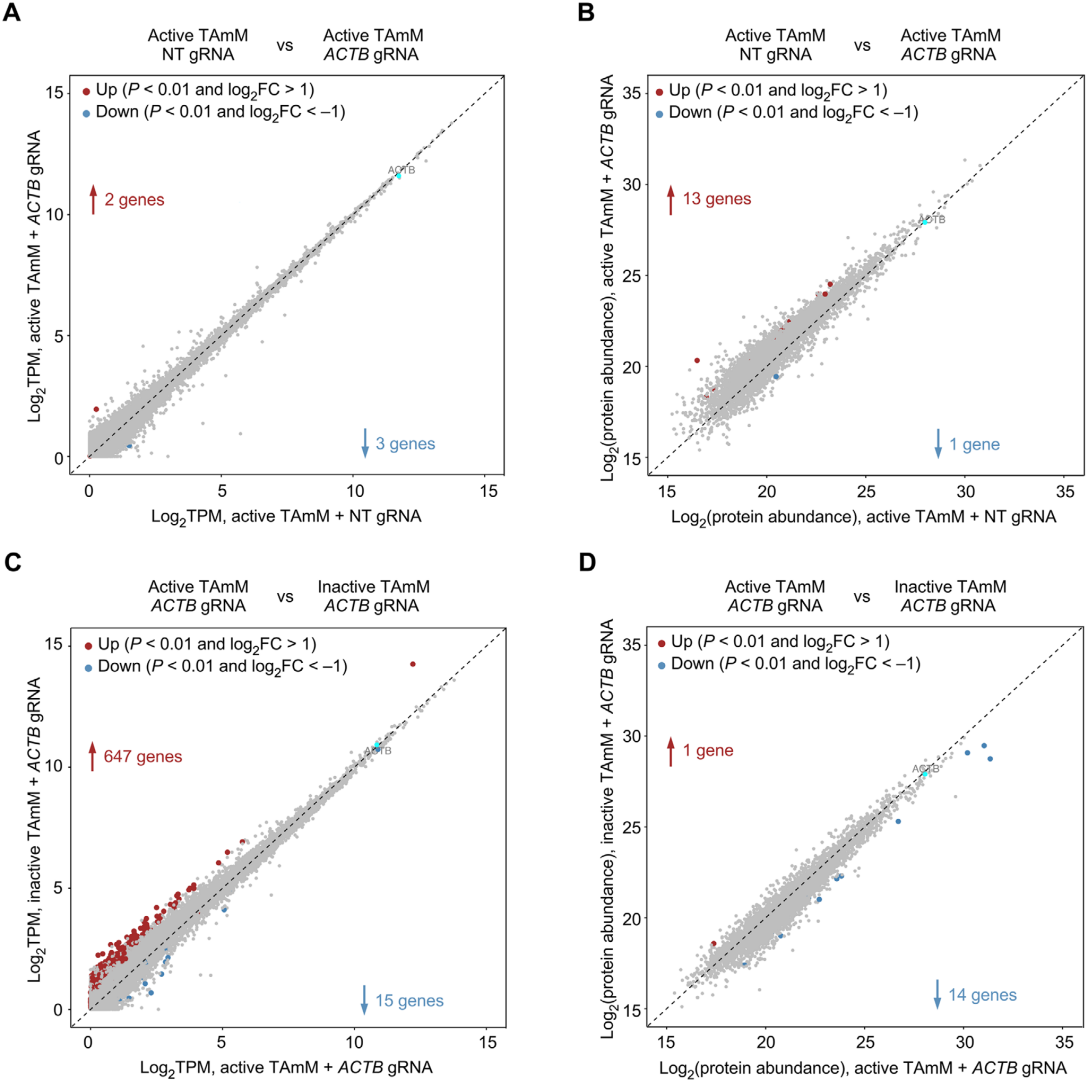
Collateral effects analysis of TAmM‐mediated m^6^Am editing on *ACTB*. (A, B) Scatter plots showing differential RNA (A) and protein (B) expression levels between TAmM‐mediated m^6^Am installation using *ACTB* gRNA versus NT gRNA. (C, D) Scatter plots displaying differential RNA (C) and protein (D) expression levels between active and inactive TAmM‐mediated m^6^Am installation using *ACTB* gRNA. n = 3 for each group. NT, non‐targeting control.

To evaluate the collateral effects of targeted m^6^Am installation mediated by TAmM, we performed transcriptome‐wide RNA sequencing (RNA‐seq) and data‐independent acquisition (DIA) mass spectrometry on HEK293T cells co‐transfected with either the active or inactive TAmM editor. Overall, co‐expression of the TAmM with *ACTB* gRNA resulted in minimal off‐target transcript degradation at both the RNA and protein levels. Specifically, only two genes were significantly downregulated and three were upregulated at the RNA level, while 13 genes were downregulated and one was upregulated at the protein level compared to the non‐targeting gRNA control (Figure 5A,B). Moreover, compared to MTase‐inactive TAmM controls, we observed 647 upregulated and 15 downregulated differentially expressed RNAs with the active TAmM editor. At the protein level, targeted m^6^Am installation by the TAmM editor resulted in one increased and 14 decreased proteins (Figure 5C,D). We also evaluated the dynamic transcriptomic and proteomic changes with a *GAPDH*‐targeting gRNA. Co‐transfection of the TAmM editor with *GAPDH* gRNA caused minimal off‐target degradation at both RNA and protein levels. Specifically, five genes were downregulated at the RNA level, while two were downregulated and 37 were upregulated at the protein level compared to the non‐targeting gRNA control (Figure S6A,B). Compared to the inactive TAmM, 547 genes were increased and 11 decreased in RNA levels, while only one protein was downregulated (Figure S6C,D). These results indicate that TAmM‐mediated m^6^Am installation caused minimal off‐target effects, further confirming the specificity and efficiency of the TAmM editor.

### 3.5 Duration‐Dependent Effects and Global Specificity of TAmM Expression

An important consideration for Cas13‐methyltransferase fusion systems is whether prolonged expression leads to progressive, nonspecific RNA methylation, given that PCIF1 retains catalytic activity independent of RNA targeting. To directly assess the global impact of sustained TAmM expression, we performed transcriptomic and proteomic analyses comparing cells expressing PCIF1‐dCasRx with a NT gRNA to mock controls, and, in parallel, cells overexpressing PCIF1 alone to mock controls (Figure S7A‐E). RNA‐seq analysis revealed that expression of PCIF1‐dCasRx with a non‐targeting gRNA resulted in substantially fewer differentially expressed genes than direct PCIF1 overexpression (Figure S7A,B). While PCIF1 overexpression caused widespread transcriptional perturbation, PCIF1‐dCasRx induced a markedly attenuated response, indicating that Cas13 fusion effectively constrains PCIF1 activity and prevents progressive, transcriptome‐wide effects during sustained expression. Consistent with this observation, pathway enrichment analysis of the limited set of genes affected by PCIF1‐dCasRx revealed predominant enrichment for immune‐ and interferon‐related pathways (Figure S7C), a well‐documented and system‐intrinsic response associated with Cas13‐/dCas13‐based RNA‐targeting platforms rather than RNA methylation per se.

To evaluate global protein‐level consequences, we next performed DIA proteomics. Compared with PCIF1 overexpression, which resulted in extensive proteome remodeling, expression of PCIF1‐dCasRx with an NT gRNA induced significantly fewer changes in protein abundance (Figure S7D,E). These proteomics results indicate that untargeted PCIF1 activity does not accumulate over time to a degree sufficient to broadly alter protein output, further supporting the high specificity of the TAmM system under sustained expression conditions. Together, these data demonstrate that although duration of expression is an important consideration for Cas13‐based RNA editors, the dCas13‐guided context substantially restricts PCIF1 activity and prevents progressive, nonspecific m^6^Am installation. This behavior is consistent with prior reports of Cas13‐directed RNA methylation systems and supports the conclusion that TAmM enables sustained yet highly specific m^6^Am editing with minimal global perturbation, representing a significant improvement over untargeted enzyme overexpression.

### 3.6 Editing Window of the TAmM System

We investigated the editing window of the TAmM editor. Since the combination of 5´ cap and Am serves as the obligatory substrate for PCIF1, we designed four gRNAs positioned 1, 8, 15, and 22 nt 3´ to the first adenosine of *ACTB* mRNA, and then measured their efficiency in directing m^6^Am installation with the TAmM editor. We observed successful m^6^Am installation into *ACTB* mRNA when TAmM was co‐transfected with each of the four gRNAs, with the gRNA ending at 8–15 nt 3´ to the first adenosine showing the highest efficiency (Figure S8). These results suggest that the most efficient targeting sites of the TAmM editor are around 10 nt 3´ to the first adenosine of target mRNAs.

### 3.7 Targeted m^6^Am Installation at *CTNNB1* Enhances Translation and Promotes Oncogenic Phenotypes

**FIGURE 6:**
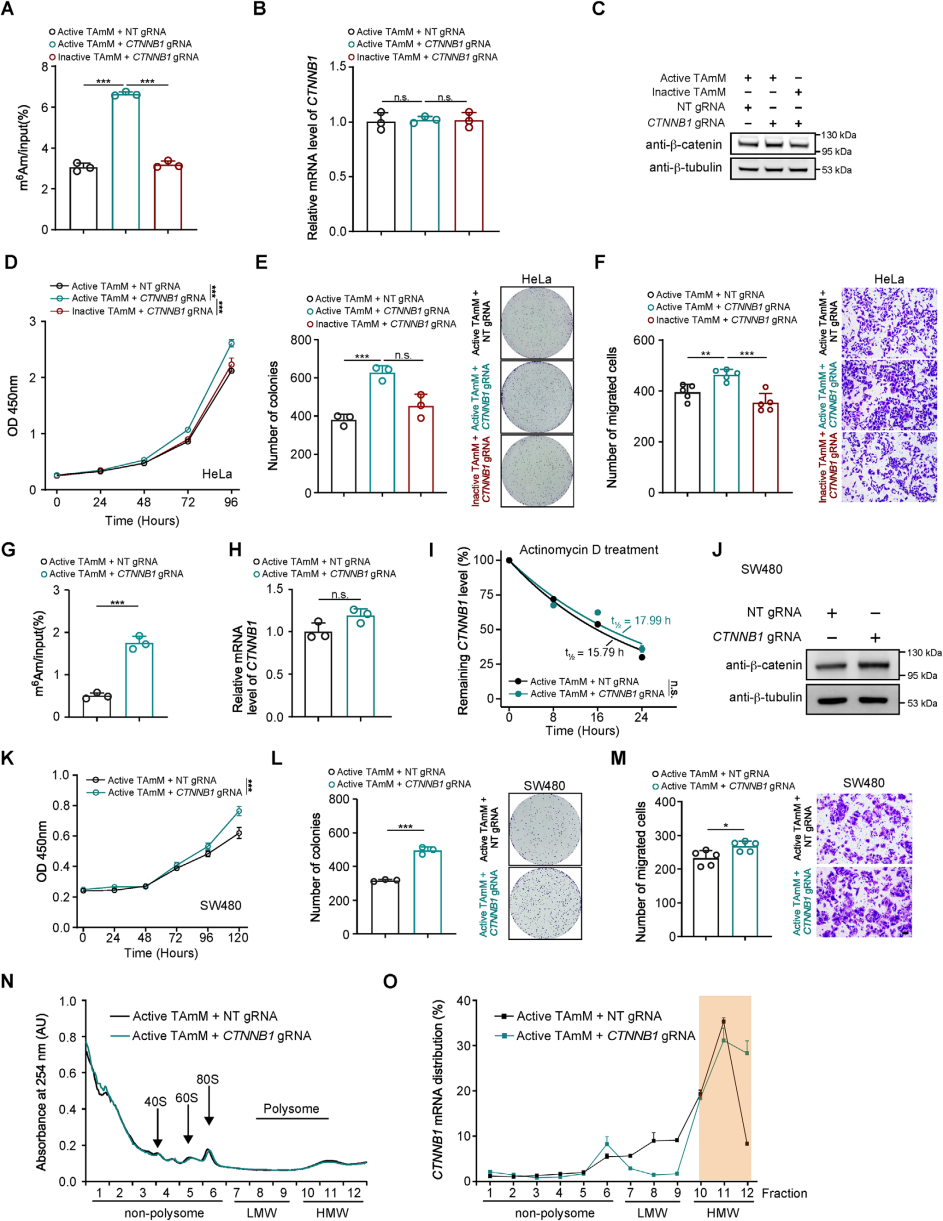
Site‐specific m^6^Am installation at *CTNNB1* enhances translation and promotes oncogenic phenotypes. (A) m^6^Am‐RIP–RT‐qPCR analysis showing increased m^6^Am enrichment at *CTNNB1* mRNA in cells expressing active TAmM with *CTNNB1* gRNA compared with NT gRNA and catalytically inactive TAmM controls. (B) RT‐qPCR analysis of *CTNNB1* mRNA abundance under the indicated conditions, showing no significant change following targeted m^6^Am installation. (C) Immunoblot analysis of *β*‐catenin protein levels in HeLa cells expressing active or inactive TAmM with NT or *CTNNB1* gRNA; *β*‐tubulin serves as a loading control. (D) Cell proliferation assay (CCK‐8) in HeLa cells demonstrating enhanced growth upon targeted m^6^Am editing of *CTNNB1*. (E) Colony formation assays and quantification showing increased clonogenic potential in HeLa cells following *CTNNB1* m^6^Am installation. Representative images are shown on the right. (F) Transwell migration assays and quantification in HeLa cells showing increased migratory capacity upon targeted *CTNNB1* m^6^Am editing; representative images are shown on the right. (G) m^6^Am‐RIP–RT‐qPCR analysis confirming increased m^6^Am enrichment at *CTNNB1* mRNA in SW480 cells expressing active TAmM with *CTNNB1* gRNA. (H) RT‐qPCR analysis showing unchanged *CTNNB1* mRNA levels in SW480 cells following targeted m^6^Am installation. (I) mRNA stability analysis of *CTNNB1* following actinomycin D treatment, indicating comparable transcript half‐lives between control and m^6^Am‐edited conditions. (J) Immunoblot analysis of β‐catenin protein levels in SW480 cells expressing NT or *CTNNB1* gRNA; *β*‐tubulin serves as a loading control. (K) CCK‐8 assay in SW480 cells showing increased growth following *CTNNB1* m^6^Am editing. (L) Colony formation assays and quantification in SW480 cells demonstrating enhanced clonogenic growth upon targeted *CTNNB1* m^6^Am installation; representative images are shown on the right. (M) Transwell migration assays and quantification in SW480 cells showing increased migratory capacity following *CTNNB1* m^6^Am editing; representative images are shown on the right. (N) Polysome profiling analysis showing global ribosome distribution in cells expressing active TAmM with NT or *CTNNB1* gRNA; positions of 40S, 60S, and 80S ribosomal subunits are indicated. (O) Distribution of *CTNNB1* mRNA across polysome fractions, demonstrating increased association with heavy polysomes (HMW) following targeted m^6^Am installation, consistent with enhanced translational efficiency. Data are presented as mean ± s.e.m. Statistical significance was determined using two‐tailed Student's *t*‐test or one‐way ANOVA, as appropriate. *P* values are indicated (* *p* < 0.05; ** *p* < 0.01; *** *p* < 0.001; n.s., not significant). NT, non‐targeting control. Scale bars, 100 µm.

To investigate whether targeted m^6^Am editing could elicit functional consequences at the level of individual transcripts, we focused on *CTNNB1* mRNA, a well‐characterized oncogene that exhibits low basal levels of cap‐adjacent m^6^Am methylation. Analysis of publicly available datasets revealed minimal endogenous m^6^Am enrichment at the *CTNNB1* TSS, indicating that *CTNNB1* is largely unmethylated under basal conditions and thus represents an ideal substrate for site‐specific m^6^Am installation (Figure S9). We next applied the TAmM system to selectively install m^6^Am at the cap‐proximal nucleotide of *CTNNB1* mRNA. Targeted m^6^Am editing was first validated by m^6^Am‐RIP–RT‐qPCR, which showed a significant increase in m^6^Am enrichment at *CTNNB1* transcripts in cells expressing active TAmM with *CTNNB1* gRNA, but not in NT gRNA or catalytically inactive TAmM controls (Figure 6A). Importantly, targeted m^6^Am installation did not alter *CTNNB1* mRNA abundance, indicating that the modification does not affect transcript levels (Figure 6B).

Despite unchanged mRNA levels, targeted m^6^Am installation resulted in a marked increase in *β*‐catenin protein abundance, as detected by immunoblotting (Figure 6C), suggesting post‐transcriptional regulation. Functional assays in HeLa cells revealed that m^6^Am‐modified *CTNNB1* significantly enhanced cell proliferation (Figure 6D), clonogenic growth (Figure 6E), and migratory capacity (Figure 6F), whereas these effects were abolished in cells expressing catalytically inactive TAmM. Consistent results were observed in SW480 colorectal cancer cells, where targeted m^6^Am installation similarly increased m^6^Am enrichment at *CTNNB1* without affecting mRNA abundance (Figure 6G,H), but significantly elevated *β*‐catenin protein levels (Figure 6J). Correspondingly, m^6^Am‐edited *CTNNB1* promoted cell proliferation (Figure 6K), colony formation (Figure 6L), and migration (Figure 6M), demonstrating that this regulatory mechanism is conserved across distinct cellular contexts.

To elucidate the molecular mechanism underlying these phenotypes, we examined *CTNNB1* mRNA stability following actinomycin D treatment and found no significant difference in transcript half‐life between control and m^6^Am‐edited conditions (Figure 6I), indicating that m^6^Am does not enhance *CTNNB1* mRNA stability. In contrast, polysome profiling revealed that m^6^Am‐modified *CTNNB1* mRNA exhibited increased association with heavy polysome fractions (Figure 6N,O), consistent with enhanced translational efficiency. Together, these data demonstrate that site‐specific m^6^Am installation at the cap‐proximal nucleotide of *CTNNB1* selectively enhances translation without affecting mRNA abundance or stability, thereby promoting *β*‐catenin–dependent proliferative and migratory phenotypes. This functional dissection provides direct evidence that individual m^6^Am sites can exert distinct and biologically meaningful regulatory roles in gene expression, underscoring the utility of TAmM for precise interrogation of m^6^Am function at endogenous transcripts.

## 4 Discussion

In this study, we expand the toolkit of epitranscriptome engineering by establishing TAmM, a programmable molecular platform capable of precisely manipulating RNA m^6^Am modifications at single‐transcript resolution. By coupling the RNA‐targeting specificity of catalytically inactive Cas13 with the m^6^Am methyltransferase PCIF1, TAmM enables direct installation of m^6^Am at defined endogenous mRNAs without substantially perturbing the global m^6^Am landscape, transcriptome, or proteome. These properties address a longstanding limitation in the field and provide a robust framework for causal interrogation of individual m^6^Am modifications.

A central advance of TAmM lies in its high specificity and minimal off‐target activity, even under sustained expression. Although PCIF1 is catalytically active independent of Cas13, our genome‐wide transcriptomic and proteomic analyses demonstrate that Cas13‐mediated RNA targeting effectively constrains PCIF1 activity, resulting in markedly fewer global perturbations compared with untargeted PCIF1 overexpression. The limited transcriptional changes observed are largely enriched for immune‐ and interferon‐related pathways, a well‐documented response intrinsic to Cas13‐based RNA‐targeting systems, rather than evidence of progressive, nonspecific m6Am accumulation. These findings underscore that prolonged expression of TAmM does not lead to cumulative off‐target methylation and highlight the importance of spatial confinement in achieving durable and precise RNA modification. Beyond tool development, our work provides direct functional insights into the biological roles of m^6^Am. By selectively installing m^6^Am at the cap‐proximal nucleotide of *CTNNB1* mRNA, we demonstrate that individual m^6^Am sites can exert transcript‐specific regulatory effects. Targeted m^6^Am editing enhances *β*‐catenin protein expression without altering mRNA abundance or stability, indicating a primary role in translational control. Polysome profiling further reveals increased association of m^6^Am‐modified *CTNNB1* mRNA with heavy polysomes, providing mechanistic evidence that cap‐adjacent m^6^Am promotes translational efficiency. Functionally, this targeted modification drives enhanced cell proliferation, clonogenicity, and migration across multiple cell types, directly linking a single m^6^Am site to oncogenic phenotypes.

These findings highlight the multifaceted nature of m^6^Am in RNA metabolism and gene regulation. While m^6^Am has been implicated in mRNA stability and translation, prior studies largely relied on global perturbation of m^6^Am writers or erasers, confounding interpretation of site‐specific effects. TAmM overcomes this limitation by enabling precise manipulation of individual m^6^Am sites, thereby revealing that the functional consequences of m^6^Am are highly context‐ and transcript‐dependent. This precision will be essential for dissecting how m^6^Am integrates with other cap‐associated features and RNA‐binding proteins to fine‐tune gene expression programs. Looking forward, the TAmM platform offers broad opportunities for studying the physiological and pathological roles of m^6^Am across diverse biological contexts, including development, stress responses, and cancer. With further optimization to enhance editing efficiency and minimize residual off‐target effects, this strategy could be extended to other emerging RNA modifications, such as *N* ^4^‐acetylcytidine (ac4C) and RNA‐associated *N*‐glycans [47, 48]. Together with complementary RNA‐targeting tools, TAmM represents a powerful approach for advancing mechanistic understanding of the epitranscriptome and for uncovering causal links between RNA modifications and cellular phenotypes.

## Author Contributions

Y.L., Q.S., and J.Z. conceived this project. Y.L., Q.S., Ya.L., J.Z., and X.T. designed experiments. Y.L., X.T., Y.H., P.W., J.Z., G.M., Ya.L., B.Y., and Q.S. performed all the experiments. Y.L., J.Z., M.G., and Q.S. analyzed the data. Y.L. and Q.S. supervised the project. Y.L. and Q.S. wrote the manuscript with input from all authors.

## Conflicts of Interest

The authors declare no conflicts of interest.

## Supporting information




**Supporting File**: advs74815‐sup‐0001‐SuppMat.docx

## Data Availability

The data that support the findings of this study are openly available in Genome Sequence Archive at https://ngdc.cncb.ac.cn/gsa‐human, reference number HRA016131, HRA008405, and HRA011540.
