# Supplementary material for: Programmable RNA N 6,2´‐O‐Dimethyladenosine Editing
Source: Adv Sci (Weinh). 2026 Mar 13;13(29):e74815. doi: 10.1002/advs.74815 (PMC13205690; doi:10.1002/advs.74815)
Supplement: Supplementary file 1 — Supporting File: advs74815‐sup‐0001‐SuppMat.docx [file ADVS-13-e74815-s001.docx]

**Supporting Information for**

Programmable RNA *N*^6^,2´-O-Dimethyladenosine Editing

Yang Li^1, 8, *^, Xiangmin Tan^2, 8^, Yaran Liu^3, 8^, Yongquan He^1^, Bo Yuan^4,5^, Ping Wang^2^, Guangzhi Ma^6^, Mengzhe Guo^7, *^, Jian Zhou^6, *^, Qiang Sun^2, *^

^1^Genetic Diseases Key Laboratory of Sichuan Province, Department of Medical Genetics, Department of Laboratory Medicine, Sichuan Academy of Medical Sciences & Sichuan Provincial People’s Hospital, School of Medicine, University of Electronic Science and Technology of China, Chengdu, China

^2^Center for RNA Medicine, the Fourth Affiliated Hospital of School of Medicine, and International School of Medicine, International Institutes of Medicine, Zhejiang University, Yiwu, China

^3^Institute of Medical Artificial Intelligence, Binzhou Medical College, Yantai, China

^4^Institute of Artificial Intelligence, Beihang University, Haidian, Beijing, China

^5^Beijing Advanced Innovation Center for Future Blockchain and Privacy Computing, Beihang University, Haidian, Beijing, China

^6^Department of Thoracic Surgery and Institute of Thoracic Oncology, West China Hospital, Sichuan University, Chengdu, China

^7^School of Pharmacy, Xuzhou Medical University, Xuzhou, China

^8^These authors contributed equally.

* To whom correspondence should be addressed: Yang Li, Mengzhe Guo, Jian Zhou, Qiang Sun

**Email:** [younglee@zju.edu.cn](mailto:younglee@zju.edu.cn(Y) (Y. L.), [guomengzhe@xzhmu.edu.cn](mailto:guomengzhe@xzhmu.edu.cn) (M. G.), [jian_zhou@wchscu.cn](mailto:jian_zhou@wchscu.cn) (J. Z.), [qsun95@zju.edu.cn](mailto:qsun95@zju.edu.cnQ) (Q. S.)

**This file includes:**

Figures S1 to S9

Tables S1 to S2

Supplemental sequences 1 to 4


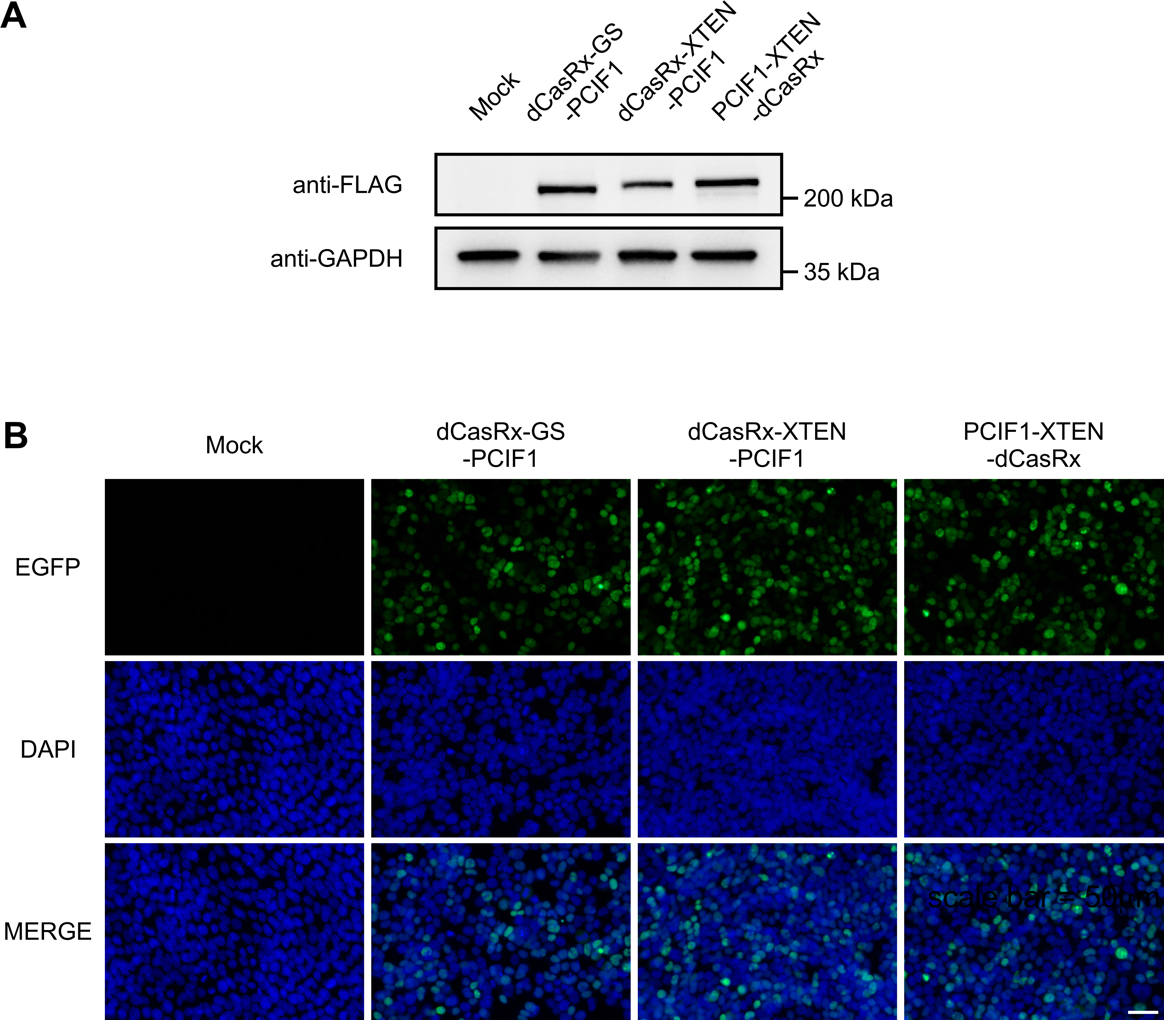


Figure S1. Expression and localization of dCasRx and PCIF1 fusions. HEK293T cells were transfected with indicated constructs and subjected to the following experiments. (A) Examination of protein expression levels of dCasRx-GS-PCIF1, dCasRx-XTEN-PCIF1, and PCIF1-XTEN-dCasRx fusions. (B) Representative immunofluorescence images showing the nuclear localization of three fusion proteins. Scale bar = 50 μm.


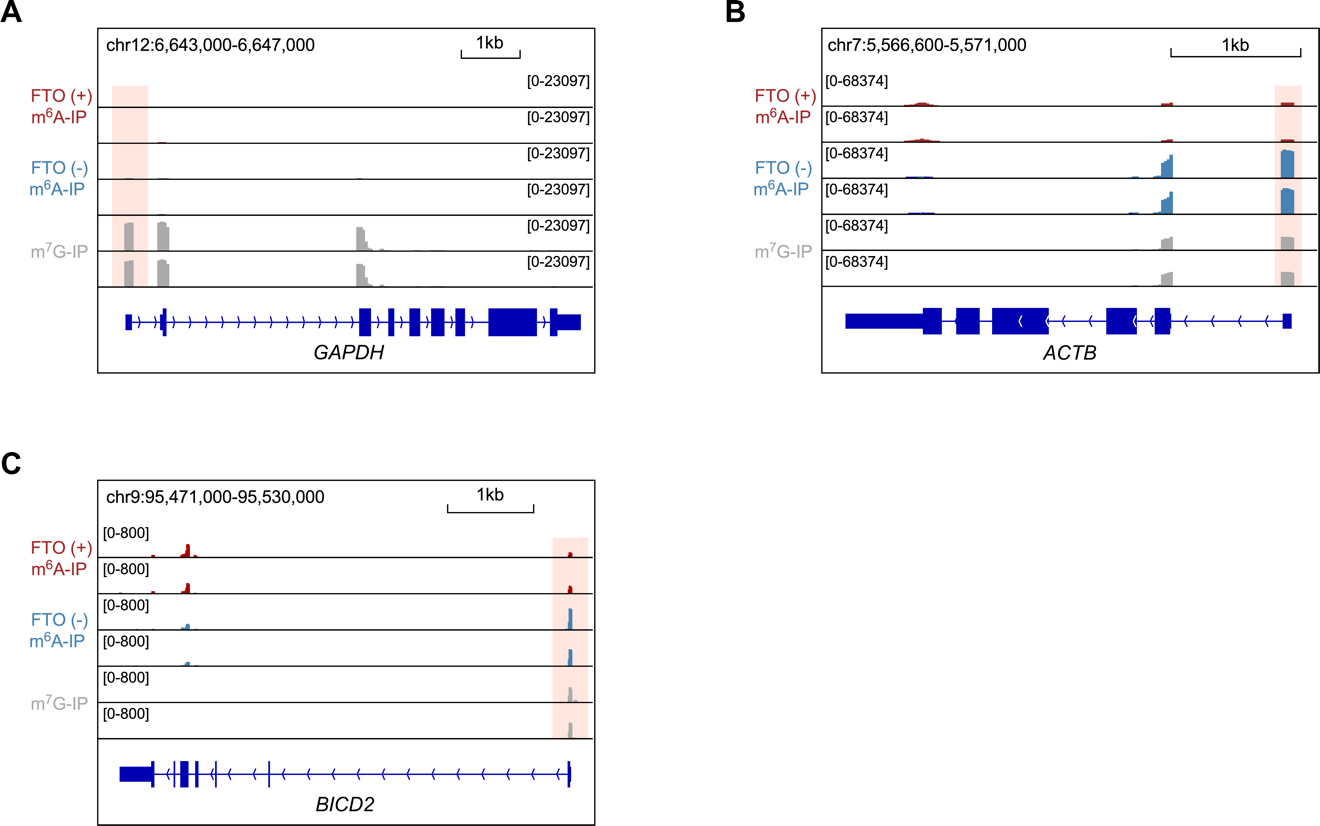


Figure S2. Representative views of typical m^6^Am peaks on *GAPDH* (A), *ACTB* (B), and *BICD2* (C)mRNAs in HEK293T cells according to m^6^Am-seq data under the accession GSE180253.


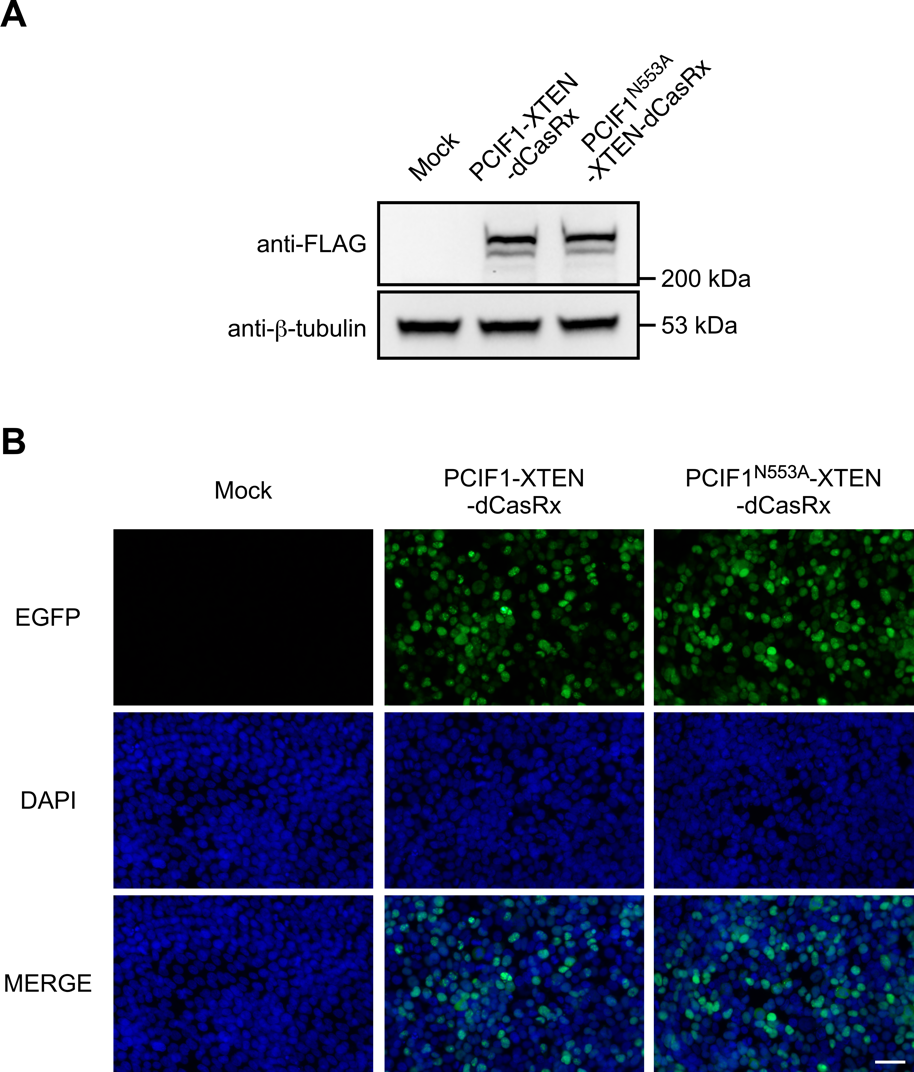


Figure S3. Expression and localization of active and inactive TAmM editors. HEK293T cells were transfected with indicated constructs and subjected to the following experiments. (A) Examination of protein expression levels of PCIF1-XTEN-dCasRx and PCIF1^N553A^-XTEN-dCasRx fusions. (B) Representative immunofluorescence images showing the nuclear localization of two fusion proteins. Scale bar = 50 μm.


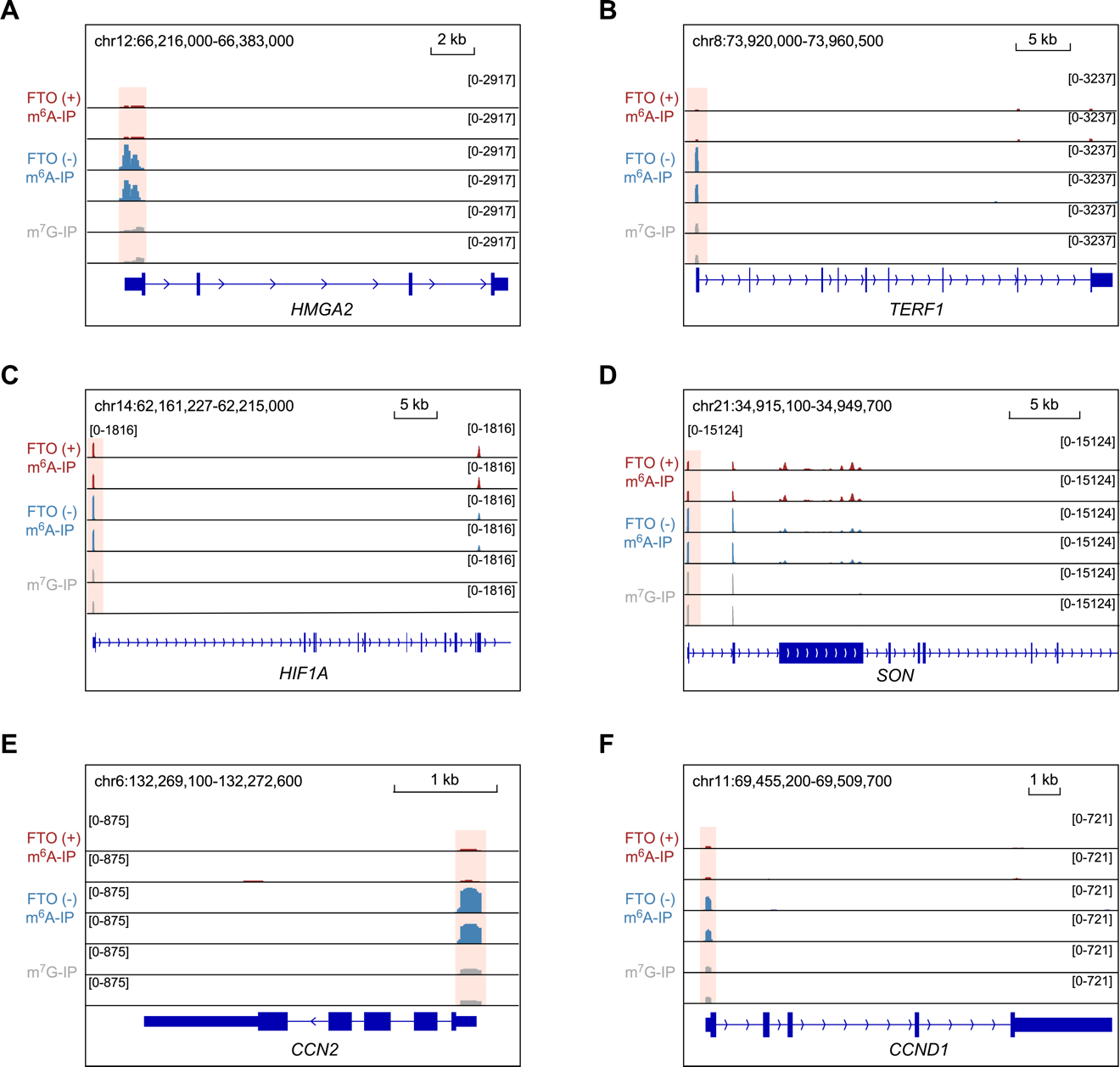


Figure S4. Representative views of typical m^6^Am peaks on potential off-target mRNAs in HEK293T cells according to m^6^Am-seq data under the accession GSE180253, including *HMGA2* (A), *TERF1* (B), *HIF1A* (C), *SON* (D), *CCN2* (E), and *CCND1* (F).


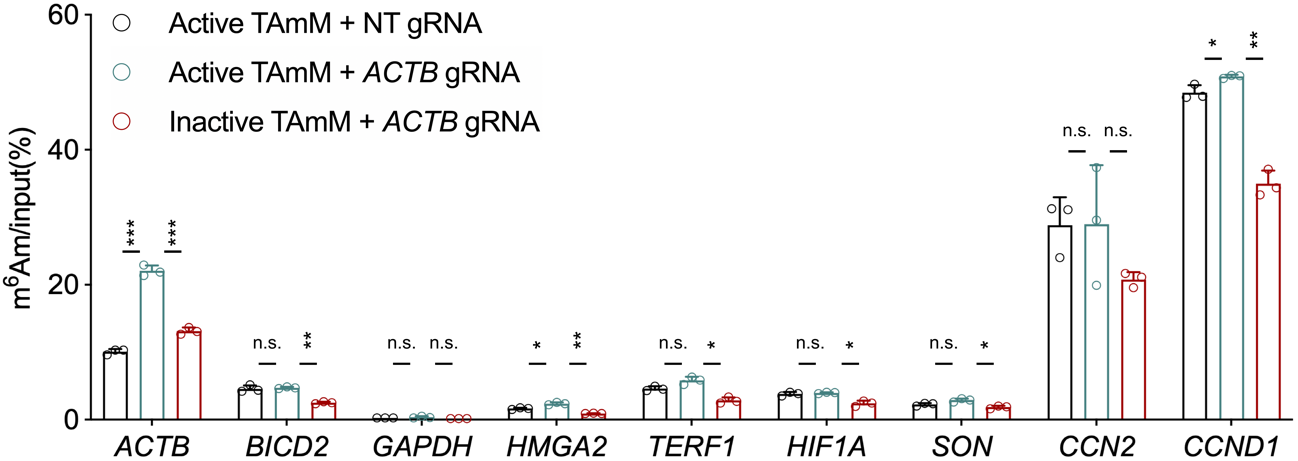


Figure S5. Specificity of the TAmM editor on *ACTB* mRNA. HEK293T cells were transfected with indicated constructs and subjected to m^6^Am-RIP-RT-qPCR. Potential off-target mRNAs (*BICD2*, *GAPDH*, *HMGA2*, *TERF1*, *HIF1A*, *SON*, *CCN2*, and *CCND1*) were quantified with specific primers targeting the corresponding 5´UTR. NT, non-targeting control. Error bars represent the mean ± s.d. (n.s., not significant, **P* < 0.05, ***P* < 0.01, ****P* < 0.001, two-tailed Student’s *t*-test).


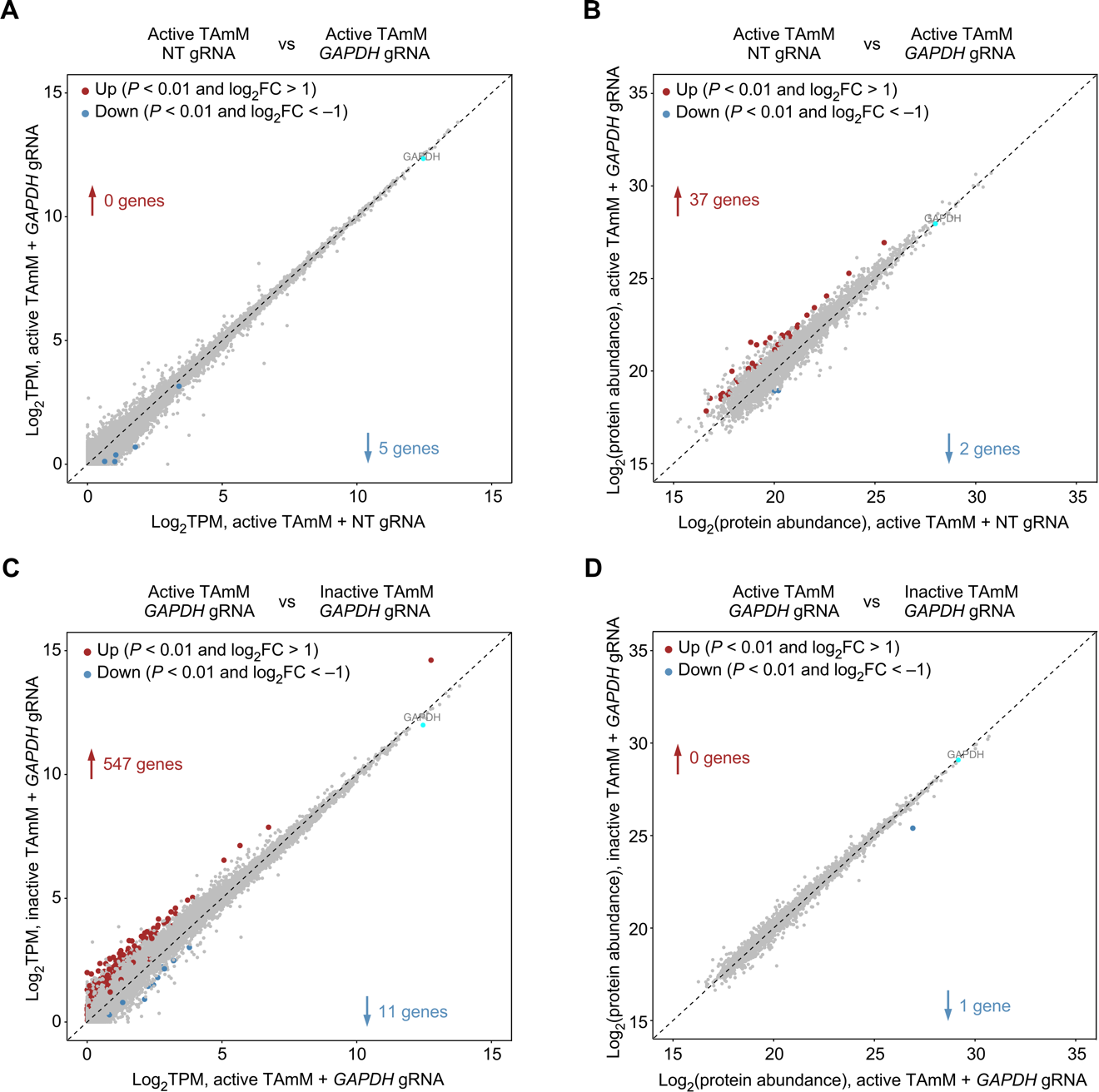


Figure S6. Collateral effect analysis of TAmM-mediated m^6^Am editing on *GAPDH*. (A and B) Scatter plots showing differential RNA (A) and protein (B) expression levels between TAmM-mediated m^6^Am installation using *GAPDH* gRNA versus NT gRNA. (C and D) Scatter plots displaying differential RNA (C) and protein (D) expression levels between active and inactive TAmM-mediated m^6^Am installation using *GAPDH* gRNA. n = 3 for each group. NT, non-targeting control.


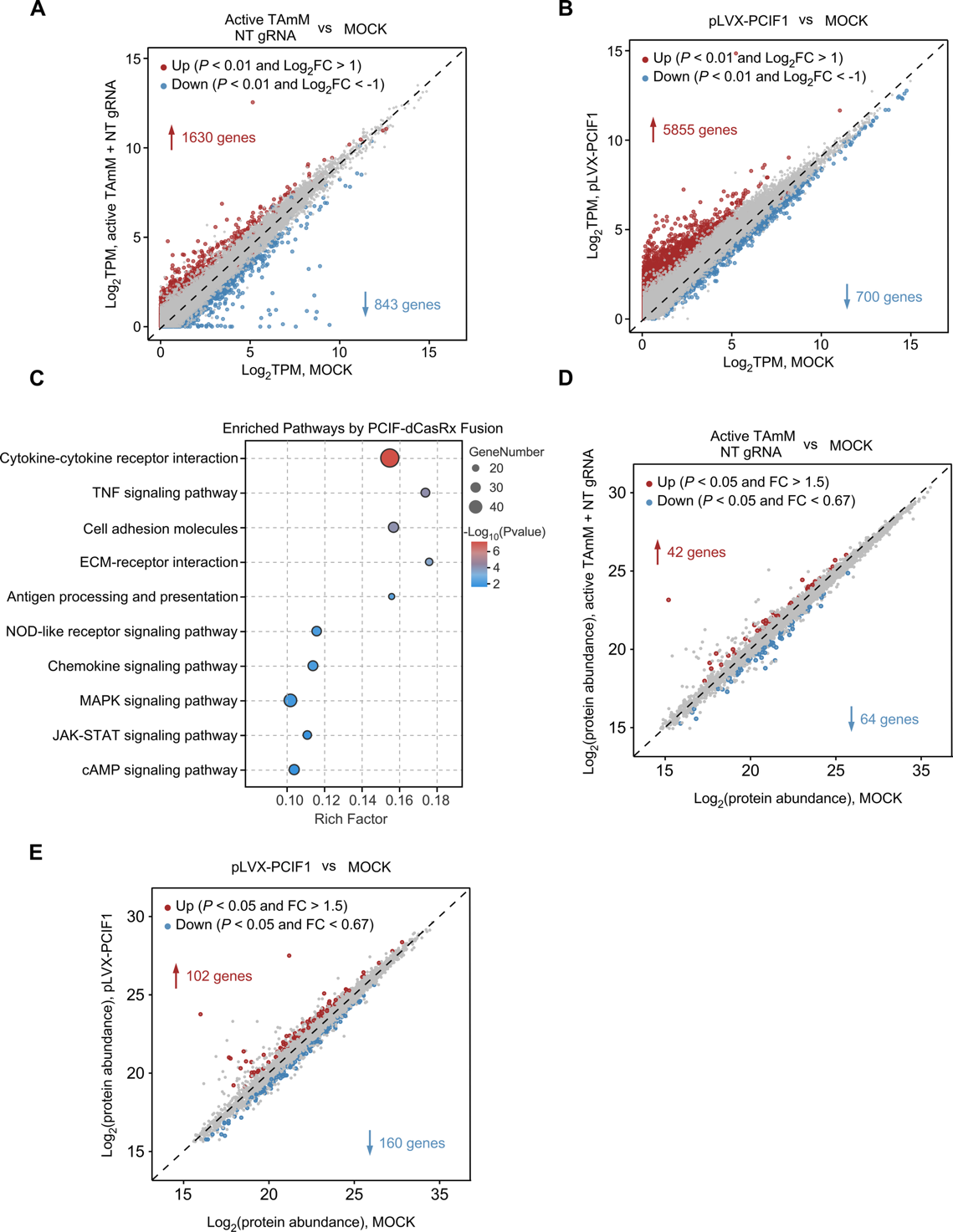


Figure S7. Global transcriptomic and proteomic analyses reveal minimal off-target effects of TAmM expression. (A) RNA-seq scatter plot comparing transcript abundance between HEK293T cells expressing active TAmM with non-targeting (NT) gRNA and mock cells. (B) RNA-seq scatter plot comparing transcript abundance between cells overexpressing PCIF1 alone (pLVX-PCIF1) and mock-treated cells, showing substantially more global transcriptional changes relative to the TAmM condition. (C) Pathway enrichment analysis of DEGs identified in cells expressing PCIF1-dCas13 with NT gRNA, revealing enrichment of immune- and interferon-related pathways characteristic of Cas13-based RNA-targeting systems. (D) Data-independent acquisition mass spectrometry (DIA-MS) scatter plot comparing global protein abundance between cells expressing active TAmM with NT gRNA and mock cells. (E) DIA-MS scatter plot comparing global protein abundance between cells overexpressing PCIF1 alone and mock cells, demonstrating substantially greater proteome perturbation than observed with TAmM expression.


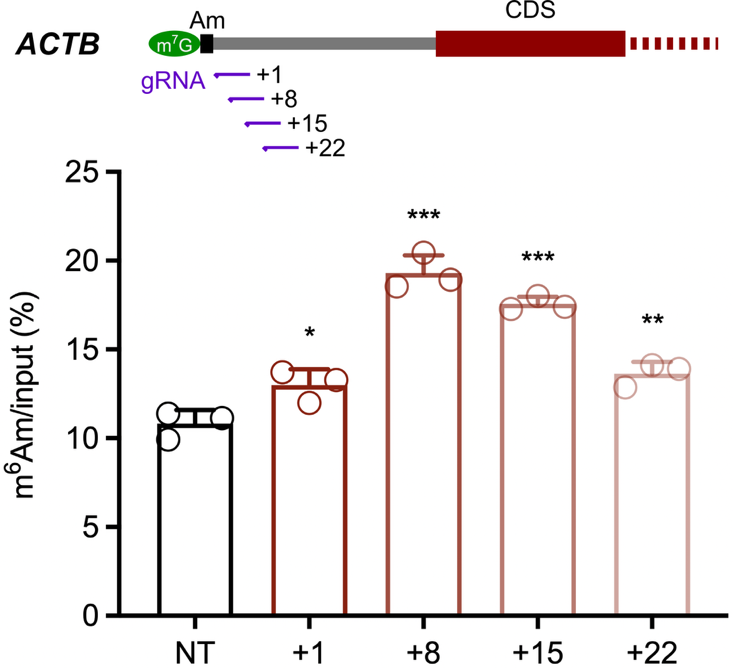


Figure S8. Characterization of the TAmM editing window at the *ACTB* transcription start site. Schematic illustration of gRNA target positions relative to the cap-adjacent adenosine (+1) of *ACTB* mRNA (top). gRNAs were designed to target positions +1, +8, +15, or +22 downstream of the transcription start site. m^6^Am-RIP-RT-qPCR quantification (bottom) shows m^6^Am enrichment at ACTB following TAmM editing with the indicated gRNAs. Targeting positions proximal to the cap (+1 to +15) resulted in robust m^6^Am installation, with maximal editing efficiency observed at +8, whereas targeting at +22 led to reduced editing efficiency. NT, non-targeting gRNA. Data are presented as mean ± s.e.m. Statistical significance was determined by one-way ANOVA compared with NT control (* *P* < 0.05; ** *P* < 0.01; *** *P* < 0.001).


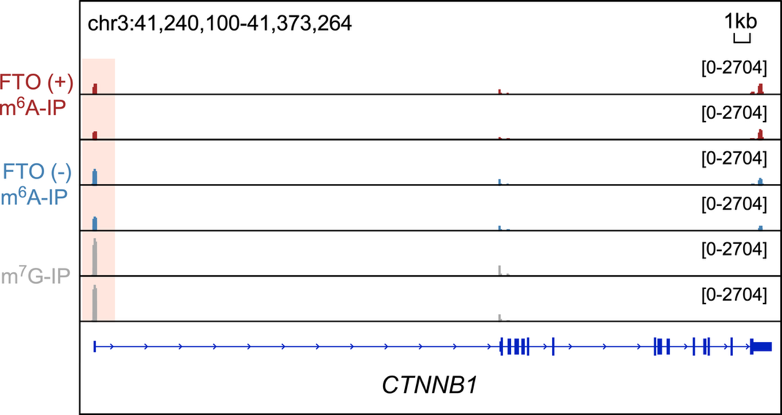


Figure S9. Representative views of *CTNNB1* m^6^Am peaks on potential off-target mRNAs in HEK293T cells according to m^6^Am-seq data under the accession GSE180253.

| **Name** | **Sequences (5´-3´)** | **Usage** | **Source** |
| --- | --- | --- | --- |
| NT gRNA | GTAATGCCTGGCTTGTCGACGCATAGTCTG | Non-targeting control | Figure 1, 2, 3, 4, 5, 6, S5, S6, S7, and S8 |
| *GAPDH* gRNA | GCTCGGCTGGCGACGCAAAAGAAGATGCGG | Targeting 5´UTR of *GAPDH* | Figure 1, 2, and S6 |
| *ACTB* gRNA | GGCGGCGGATCGGCAAAGGCGAGGCTCTGT | Targeting 5´UTR of *ACTB* | Figure 1, 2, 3, 4, 5, S5, and S8 |
| *BICD2* gRNA | GACGGCGCCGACATGGTGGCCGAGGGCTGA | Targeting 5´UTR of *BICD2* | Figure 2 and S6 |
| *CTNNB1* gRNA | CGGGGCCGGGCCAACGCTGCTGCCACAGAC | Targeting 5´UTR of *CTNNB1* | Figure 6 |

Table S1. Guide RNA spacer sequences used in this study.

| **Name** | **Sequences (5´-3´)** | **Usage** | **Source** |
| --- | --- | --- | --- |
| q*GAPDH*-F | GACAGTCAGCCGCATCTTCT | m^6^Am-RIP-RT-qPCR | Figure 1, 2, and S5 |
| q*GAPDH*-R | GCGCCCAATACGACCAAATC | m^6^Am-RIP-RT-qPCR | Figure 1, 2 and S5 |
| q*ACTB*-F | ACAGAGCCTCGCCTTTGCCGAT | m^6^Am-RIP-RT-qPCR | Figure 1, 2, and S5 |
| q*ACTB*-R | CGGCGATATCATCATCCATG | m^6^Am-RIP-RT-qPCR | Figure 1, 2, and S5 |
| q*BICD2*-F | TGCGAGAGCCTCAGTGGGAG | m^6^Am-RIP-RT-qPCR | Figure 2 and S5 |
| q*BICD2*-R | ATCACCAGCCGCGCGTACTCCT | m^6^Am-RIP-RT-qPCR | Figure 2 and S5 |
| q*CTNNB1*-F | TCTGAGGAGCAGCTTCAGTC | m^6^Am-RIP-RT-qPCR | Figure 6 |
| q*CTNNB1*-R | ATACTTCAAATACCCTCAGG | m^6^Am-RIP-RT-qPCR | Figure 6 |
| q*HMGA2*-F | AAGACTCAGGAGCTAGCAGC | m^6^Am-RIP-RT-qPCR | Figure S5 |
| q*HMGA2*-R | AGGCAGAGGACAGAGTAGTG | m^6^Am-RIP-RT-qPCR | Figure S5 |
| q*TERF1*-F | AGCCATTTAACATGGCGGAG | m^6^Am-RIP-RT-qPCR | Figure S5 |
| q*TERF1*-R | TCTCTGTTTCTGCCATCTGC | m^6^Am-RIP-RT-qPCR | Figure S5 |
| q*HIF1A*-F | TGCACAGTGCTGCCTCGTCT | m^6^Am-RIP-RT-qPCR | Figure S5 |
| q*HIF1A*-R | CTCCTCAGGTGGCTTGTCAG | m^6^Am-RIP-RT-qPCR | Figure S5 |
| q*SON*-F | ACTAGCGAGGAGGAGTTGAG | m^6^Am-RIP-RT-qPCR | Figure S5 |
| q*SON*-R | ATTCAGCTGGCCTTCATTCC | m^6^Am-RIP-RT-qPCR | Figure S5 |
| q*CCN2*-F | CTGAGAGGAGACAGCCAGTG | m^6^Am-RIP-RT-qPCR | Figure S5 |
| q*CCN2*-R | GCTGGCGGTGGTCGGAGGTG | m^6^Am-RIP-RT-qPCR | Figure S5 |
| q*CCND1*-F | TAGCAGCGAGCAGCAGAGTC | m^6^Am-RIP-RT-qPCR | Figure S5 |
| q*CCND1*-R | GAGCTGGTGTTCCATGGCTG | m^6^Am-RIP-RT-qPCR | Figure S5 |
| q*ACTB*-Fwd | CACCATTGGCAATGAGCGGTTC | RT-qPCR | Figure 2 |
| q*ACTB*-Rev | AGGTCTTTGCGGATGTCCACGT | RT-qPCR | Figure 2 |
| q*BICD2*-Fwd | ATCCTCAAGCTGAAGTCGCTG | RT-qPCR | Figure 2 |
| q*BICD2*-Rev | CTCATTGCGCAGCTTCATCATG | RT-qPCR | Figure 2 |
| q*GAPDH*-Fwd | GGAGCGAGATCCCTCCAAAAT | RT-qPCR | Figure 2 |
| q*GAPDH*-Rev | GGCTGTTGTCATACTTCTCATGG | RT-qPCR | Figure 2 |
| q*CTNNB1*-Fwd | AGCTGACCAGCTCTCTCTTC | RT-qPCR | Figure 6 |
| q*CTNNB1*-Rev | TAGCTAGGATCATCCTGGCG | RT-qPCR | Figure 6 |
| q18S RNA-F | GGATGTAAAGGATGGAAAATACA | RT-qPCR | Figure 2 and 6 |
| q18S RNA-R | TCCAGGTCTTCACGGAGCTTGTT | RT-qPCR | Figure 2 and 6 |

Table S2. Primers used in this study.

**Supplemental sequence 1. Amino acid sequences of dCasRx-GS-PCIF1 fusions**

**NLS-dCasRx-GS-PCIF1-NLS**

MSPKKKRKVEASIEKKKSFAKGMGVKSTLVSGSKVYMTTFAEGSDARLEKIVEGDSIRSVNEGEAFSAEMADKNAGYKIGNAKFSHPKGYAVVANNPLYTGPVQQDMLGLKETLEKRYFGESADGNDNICIQVIHNILDIEKILAEYITNAAYAVNNISGLDKDIIGFGKFSTVYTYDEFKDPEHHRAAFNNNDKLINAIKAQYDEFDNFLDNPRLGYFGQAFFSKEGRNYIINYGNECYDILALLSGLAHWVVANNEEESRISRTWLYNLDKNLDNEYISTLNYLYDRITNELTNSFSKNSAANVNYIAETLGINPAEFAEQYFRFSIMKEQKNLGFNITKLREVMLDRKDMSEIRKNHKVFDSIRTKVYTMMDFVIYRYYIEEDAKVAAANKSLPDNEKSLSEKDIFVINLRGSFNDDQKDALYYDEANRIWRKLENIMHNIKEFRGNKTREYKKKDAPRLPRILPAGRDVSAFSKLMYALTMFLDGKEINDLLTTLINKFDNIQSFLKVMPLIGVNAKFVEEYAFFKDSAKIADELRLIKSFARMGEPIADARRAMYIDAIRILGTNLSYDELKALADTFSLDENGNKLKKGKHGMRNFIINNVISNKRFHYLIRYGDPAHLHEIAKNEAVVKFVLGRIADIQKKQGQNGKNQIDRYYETCIGKDKGKSVSEKVDALTKIITGMNYDQFDKKRSVIEDTGRENAEREKFKKIISLYLTVIYHILKNIVNINARYVIGFHCVERDAQLYKEKGYDINLKKLEEKGFSSVTKLCAGIDETAPDKRKDVEKEMAERAKESIDSLESANPKLYANYIKYSDEKKAEEFTRQINREKAKTALNAYLRNTKWNVIIREDLLRIDNKTCTLFANKAVALEVARYVHAYINDIAEVNSYFQLYHYIMQRIIMNERYEKSSGKVSEYFDAVNDEKKYNDRLLKLLCVPFGYCIPRFKNLSIEALFDRNEAAKFDKEKKKVSGNSGSMANENHGSPREEASLLSHSPGTSNQSQPCSPKPIRLVQDLPEELVHAGWEKCWSRRENRPYYFNRFTNQSLWEMPVLGQHDVISDPLGLNATPLPQDSSLVETPPAENKPRKRQLSEEQPSGNGVKKPKIEIPVTPTGQSVPSSPSIPGTPTLKMWGTSPEDKQQAALLRPTEVYWDLDIQTNAVIKHRGPSEVLPPHPEVELLRSQLILKLRQHYRELCQQREGIEPPRESFNRWMLERKVVDKGSDPLLPSNCEPVVSPSMFREIMNDIPIRLSRIKFREEAKRLLFKYAEAARRLIESRSASPDSRKVVKWNVEDTFSWLRKDHSASKEDYMDRLEHLRRQCGPHVSAAAKDSVEGICSKIYHISLEYVKRIREKHLAILKENNISEEVEAPEVEPRLVYCYPVRLAVSAPPMPSVEMHMENNVVCIRYKGEMVKVSRNYFSKLWLLYRYSCIDDSAFERFLPRVWCLLRRYQMMFGVGLYEGTGLQGSLPVHVFEALHRLFGVSFECFASPLNCYFRQYCSAFPDTDGYFGSRGPCLDFAPLSGSFEANPPFCEELMDAMVSHFERLLESSPEPLSFIVFIPEWREPPTPALTRMEQSRFKRHQLILPAFEHEYRSGSQHICKKEEMHYKAVHNTAVLFLQNDPGFAKWAPTPERL

QELSAAYRQSGRSHSSGSSSSSSSEAKDRDSGREQGPSREPHPTKRPAATKKAGQAKKKK

**Supplemental sequence 2. Amino acid sequences of dCasRx-XTEN-PCIF1 fusions**

**NLS-dCasRx-XTEN-PCIF1-NLS**

MSPKKKRKVEASIEKKKSFAKGMGVKSTLVSGSKVYMTTFAEGSDARLEKIVEGDSIRSVNEGEAFSAEMADKNAGYKIGNAKFSHPKGYAVVANNPLYTGPVQQDMLGLKETLEKRYFGESADGNDNICIQVIHNILDIEKILAEYITNAAYAVNNISGLDKDIIGFGKFSTVYTYDEFKDPEHHRAAFNNNDKLINAIKAQYDEFDNFLDNPRLGYFGQAFFSKEGRNYIINYGNECYDILALLSGLAHWVVANNEEESRISRTWLYNLDKNLDNEYISTLNYLYDRITNELTNSFSKNSAANVNYIAETLGINPAEFAEQYFRFSIMKEQKNLGFNITKLREVMLDRKDMSEIRKNHKVFDSIRTKVYTMMDFVIYRYYIEEDAKVAAANKSLPDNEKSLSEKDIFVINLRGSFNDDQKDALYYDEANRIWRKLENIMHNIKEFRGNKTREYKKKDAPRLPRILPAGRDVSAFSKLMYALTMFLDGKEINDLLTTLINKFDNIQSFLKVMPLIGVNAKFVEEYAFFKDSAKIADELRLIKSFARMGEPIADARRAMYIDAIRILGTNLSYDELKALADTFSLDENGNKLKKGKHGMRNFIINNVISNKRFHYLIRYGDPAHLHEIAKNEAVVKFVLGRIADIQKKQGQNGKNQIDRYYETCIGKDKGKSVSEKVDALTKIITGMNYDQFDKKRSVIEDTGRENAEREKFKKIISLYLTVIYHILKNIVNINARYVIGFHCVERDAQLYKEKGYDINLKKLEEKGFSSVTKLCAGIDETAPDKRKDVEKEMAERAKESIDSLESANPKLYANYIKYSDEKKAEEFTRQINREKAKTALNAYLRNTKWNVIIREDLLRIDNKTCTLFANKAVALEVARYVHAYINDIAEVNSYFQLYHYIMQRIIMNERYEKSSGKVSEYFDAVNDEKKYNDRLLKLLCVPFGYCIPRFKNLSIEALFDRNEAAKFDKEKKKVSGNSSGSETPGTSESATPESMANENHGSPREEASLLSHSPGTSNQSQPCSPKPIRLVQDLPEELVHAGWEKCWSRRENRPYYFNRFTNQSLWEMPVLGQHDVISDPLGLNATPLPQDSSLVETPPAENKPRKRQLSEEQPSGNGVKKPKIEIPVTPTGQSVPSSPSIPGTPTLKMWGTSPEDKQQAALLRPTEVYWDLDIQTNAVIKHRGPSEVLPPHPEVELLRSQLILKLRQHYRELCQQREGIEPPRESFNRWMLERKVVDKGSDPLLPSNCEPVVSPSMFREIMNDIPIRLSRIKFREEAKRLLFKYAEAARRLIESRSASPDSRKVVKWNVEDTFSWLRKDHSASKEDYMDRLEHLRRQCGPHVSAAAKDSVEGICSKIYHISLEYVKRIREKHLAILKENNISEEVEAPEVEPRLVYCYPVRLAVSAPPMPSVEMHMENNVVCIRYKGEMVKVSRNYFSKLWLLYRYSCIDDSAFERFLPRVWCLLRRYQMMFGVGLYEGTGLQGSLPVHVFEALHRLFGVSFECFASPLNCYFRQYCSAFPDTDGYFGSRGPCLDFAPLSGSFEANPPFCEELMDAMVSHFERLLESSPEPLSFIVFIPEWREPPTPALTRMEQSRFKRHQLILPAFEHEYRSGSQHICKKEEMHYKAVHNTAVLFLQNDPGFAKWAPTPERLQELSAAYRQ

SGRSHSSGSSSSSSSEAKDRDSGREQGPSREPHPTKRPAATKKAGQAKKKK

**Supplemental sequence 3. Amino acid sequences of PCIF1-XTEN-dCasRx fusions**

**NLS-PCIF1-XTEN-dCasRx-NLS**

MSPKKKRKVEASMANENHGSPREEASLLSHSPGTSNQSQPCSPKPIRLVQDLPEELVHAGWEKCWSRRENRPYYFNRFTNQSLWEMPVLGQHDVISDPLGLNATPLPQDSSLVETPPAENKPRKRQLSEEQPSGNGVKKPKIEIPVTPTGQSVPSSPSIPGTPTLKMWGTSPEDKQQAALLRPTEVYWDLDIQTNAVIKHRGPSEVLPPHPEVELLRSQLILKLRQHYRELCQQREGIEPPRESFNRWMLERKVVDKGSDPLLPSNCEPVVSPSMFREIMNDIPIRLSRIKFREEAKRLLFKYAEAARRLIESRSASPDSRKVVKWNVEDTFSWLRKDHSASKEDYMDRLEHLRRQCGPHVSAAAKDSVEGICSKIYHISLEYVKRIREKHLAILKENNISEEVEAPEVEPRLVYCYPVRLAVSAPPMPSVEMHMENNVVCIRYKGEMVKVSRNYFSKLWLLYRYSCIDDSAFERFLPRVWCLLRRYQMMFGVGLYEGTGLQGSLPVHVFEALHRLFGVSFECFASPLNCYFRQYCSAFPDTDGYFGSRGPCLDFAPLSGSFEANPPFCEELMDAMVSHFERLLESSPEPLSFIVFIPEWREPPTPALTRMEQSRFKRHQLILPAFEHEYRSGSQHICKKEEMHYKAVHNTAVLFLQNDPGFAKWAPTPERLQELSAAYRQSGRSHSSGSSSSSSSEAKDRDSGREQGPSREPHPTSGSETPGTSESATPESIEKKKSFAKGMGVKSTLVSGSKVYMTTFAEGSDARLEKIVEGDSIRSVNEGEAFSAEMADKNAGYKIGNAKFSHPKGYAVVANNPLYTGPVQQDMLGLKETLEKRYFGESADGNDNICIQVIHNILDIEKILAEYITNAAYAVNNISGLDKDIIGFGKFSTVYTYDEFKDPEHHRAAFNNNDKLINAIKAQYDEFDNFLDNPRLGYFGQAFFSKEGRNYIINYGNECYDILALLSGLAHWVVANNEEESRISRTWLYNLDKNLDNEYISTLNYLYDRITNELTNSFSKNSAANVNYIAETLGINPAEFAEQYFRFSIMKEQKNLGFNITKLREVMLDRKDMSEIRKNHKVFDSIRTKVYTMMDFVIYRYYIEEDAKVAAANKSLPDNEKSLSEKDIFVINLRGSFNDDQKDALYYDEANRIWRKLENIMHNIKEFRGNKTREYKKKDAPRLPRILPAGRDVSAFSKLMYALTMFLDGKEINDLLTTLINKFDNIQSFLKVMPLIGVNAKFVEEYAFFKDSAKIADELRLIKSFARMGEPIADARRAMYIDAIRILGTNLSYDELKALADTFSLDENGNKLKKGKHGMRNFIINNVISNKRFHYLIRYGDPAHLHEIAKNEAVVKFVLGRIADIQKKQGQNGKNQIDRYYETCIGKDKGKSVSEKVDALTKIITGMNYDQFDKKRSVIEDTGRENAEREKFKKIISLYLTVIYHILKNIVNINARYVIGFHCVERDAQLYKEKGYDINLKKLEEKGFSSVTKLCAGIDETAPDKRKDVEKEMAERAKESIDSLESANPKLYANYIKYSDEKKAEEFTRQINREKAKTALNAYLRNTKWNVIIREDLLRIDNKTCTLFANKAVALEVARYVHAYINDIAEVNSYFQLYHYIMQRIIMNERYEKSSGKVSEYFDAVNDEKKYNDRLLKLL

CVPFGYCIPRFKNLSIEALFDRNEAAKFDKEKKKVSGNSKRPAATKKAGQAKKKK

**Supplemental sequence 3. Amino acid sequences of PCIF1^N553A^-XTEN-dCasRx fusions**

**NLS-PCIF1^N553A^-XTEN-dCasRx-NLS** (The N553A residue is underlined and bold)

MSPKKKRKVEASMANENHGSPREEASLLSHSPGTSNQSQPCSPKPIRLVQDLPEELVHAGWEKCWSRRENRPYYFNRFTNQSLWEMPVLGQHDVISDPLGLNATPLPQDSSLVETPPAENKPRKRQLSEEQPSGNGVKKPKIEIPVTPTGQSVPSSPSIPGTPTLKMWGTSPEDKQQAALLRPTEVYWDLDIQTNAVIKHRGPSEVLPPHPEVELLRSQLILKLRQHYRELCQQREGIEPPRESFNRWMLERKVVDKGSDPLLPSNCEPVVSPSMFREIMNDIPIRLSRIKFREEAKRLLFKYAEAARRLIESRSASPDSRKVVKWNVEDTFSWLRKDHSASKEDYMDRLEHLRRQCGPHVSAAAKDSVEGICSKIYHISLEYVKRIREKHLAILKENNISEEVEAPEVEPRLVYCYPVRLAVSAPPMPSVEMHMENNVVCIRYKGEMVKVSRNYFSKLWLLYRYSCIDDSAFERFLPRVWCLLRRYQMMFGVGLYEGTGLQGSLPVHVFEALHRLFGVSFECFASPLNCYFRQYCSAFPDTDGYFGSRGPCLDFAPLSGSFEA**A**PPFCEELMDAMVSHFERLLESSPEPLSFIVFIPEWREPPTPALTRMEQSRFKRHQLILPAFEHEYRSGSQHICKKEEMHYKAVHNTAVLFLQNDPGFAKWAPTPERLQELSAAYRQSGRSHSSGSSSSSSSEAKDRDSGREQGPSREPHPTSGSETPGTSESATPESIEKKKSFAKGMGVKSTLVSGSKVYMTTFAEGSDARLEKIVEGDSIRSVNEGEAFSAEMADKNAGYKIGNAKFSHPKGYAVVANNPLYTGPVQQDMLGLKETLEKRYFGESADGNDNICIQVIHNILDIEKILAEYITNAAYAVNNISGLDKDIIGFGKFSTVYTYDEFKDPEHHRAAFNNNDKLINAIKAQYDEFDNFLDNPRLGYFGQAFFSKEGRNYIINYGNECYDILALLSGLAHWVVANNEEESRISRTWLYNLDKNLDNEYISTLNYLYDRITNELTNSFSKNSAANVNYIAETLGINPAEFAEQYFRFSIMKEQKNLGFNITKLREVMLDRKDMSEIRKNHKVFDSIRTKVYTMMDFVIYRYYIEEDAKVAAANKSLPDNEKSLSEKDIFVINLRGSFNDDQKDALYYDEANRIWRKLENIMHNIKEFRGNKTREYKKKDAPRLPRILPAGRDVSAFSKLMYALTMFLDGKEINDLLTTLINKFDNIQSFLKVMPLIGVNAKFVEEYAFFKDSAKIADELRLIKSFARMGEPIADARRAMYIDAIRILGTNLSYDELKALADTFSLDENGNKLKKGKHGMRNFIINNVISNKRFHYLIRYGDPAHLHEIAKNEAVVKFVLGRIADIQKKQGQNGKNQIDRYYETCIGKDKGKSVSEKVDALTKIITGMNYDQFDKKRSVIEDTGRENAEREKFKKIISLYLTVIYHILKNIVNINARYVIGFHCVERDAQLYKEKGYDINLKKLEEKGFSSVTKLCAGIDETAPDKRKDVEKEMAERAKESIDSLESANPKLYANYIKYSDEKKAEEFTRQINREKAKTALNAYLRNTKWNVIIREDLLRIDNKTCTLFANKAVALEVARYVHAYINDIAEVNSYFQLYHYIMQRIIMNERYEKSSGKVSEYFDAVNDEKKYNDRLLKLL

CVPFGYCIPRFKNLSIEALFDRNEAAKFDKEKKKVSGNSKRPAATKKAGQAKKKK
